# Rapid genome-wide profiling of DNA methylation and genetic variation using guide positioning sequencing (GPS)

**DOI:** 10.3389/fcell.2024.1457387

**Published:** 2024-09-24

**Authors:** Baolong Zhang, Wei Li, Jin Li, Yan Li, Huaibing Luo, Yanping Xi, Shihua Dong, Feizhen Wu, Wenqiang Yu

**Affiliations:** ^1^ Institutes of Biomedical Sciences, Shanghai Medical College, Fudan University, Shanghai, China; ^2^ Key Laboratory of Medical Epigenetics and Metabolism, Institute of Clinical Science of Zhongshan Hospital and Institutes of Biomedical Sciences, Fudan University, Shanghai, China

**Keywords:** DNA methylation, GPS, T4 DNA polymerase, genetic and epigenetic variations, protocol

## Abstract

Whole-genome bisulfite sequencing (WGBS) has been extensively utilized for DNA methylation profiling over the past decade. However, it has shown limitations in terms of high costs and inefficiencies. The productivity and accuracy of DNA methylation detection rely critically on the optimization of methodologies and the continuous refinements of related sequencing platforms. Here, we describe a detailed protocol of guide positioning sequencing (GPS), a bisulfite-based, location-specific sequencing technology designed for comprehensive DNA methylation characterization across the genome. The fundamental principle of GPS lies in the substitution of dCTP with 5-methyl-dCTP (5 mC) at the 3′-end of DNA fragments by T4 DNA polymerase, which protects cytosines from bisulfite conversion to preserve the integrity of the base composition. This alteration allows the 3′-end to independently facilitate genetic variation profiling and guides the 5′-end, enriched with methylation information, to align more rapidly to the reference genome. Hence, GPS enables the concurrent detection of both genetic and epigenetic variations. Additionally, we provide an accessible description of the data processing, specifically involving certain software and scripts. Overall, the entire GPS procedure can be completed within a maximum of 15 days, starting with a low initial DNA input of 100–500 ng, followed by 4–5 days for library construction, 8–10 days for high-throughput sequencing (HTS) and data analysis, which can greatly facilitate the promotion and application of DNA methylation detection, especially for the rapid clinical diagnosis of diverse disease pathologies associated with concurrent genetic and epigenetic variations.

## 1 Introduction

In human somatic cells, DNA methylation predominantly occurs at the 5-position of the pyrimidine ring of cytosines within approximately 29 million CpG dinucleotides across the entire haploid genome (Tse et al., 2021). Genome-wide analysis reveals that nearly 7% of CpG sites are distributed within 24,163 unique CpG islands (CGIs), primarily located in the promoter regions of 75% of all genes, with the majority (60%–80%) being methylated (Cain JA et al., 2022; Youk et al., 2020; Hernando-Herraez et al., 2015). Recent advancements in genome-wide methylation mapping techniques have revealed that 5-methylcytosine, comprising approximately 1% of all bases, demonstrates remarkable sensitivity to environmental modifications, dietary influences, and pharmacological interventions. This epigenetic mark dynamically modulates both the structural integrity and functional capacities of the genome (Bruce and Eckel, 2023; Kringel et al., 2021; Buitrago et al., 2021; Wu et al., 2023). The regulation of gene expression by local DNA methylation is intricately connected to histone modification and chromatin remodeling, processes universally implicated in both development and disease (Wang et al., 2022a; Y Yuan et al., 2023; Yang et al., 2023; Janssen and Lorincz, 2022). The dynamic transition of cytosine methylation status, mediated by DNA methyltransferases (e.g., DNMT1, DNMT3A, DNMT3B) and demethylases (e.g., the TET family), generates variable yet heritable patterns, which play roles in normal development (e.g., imprinted genes) and disease progression (e.g., tumors and metabolic diseases) (Yang et al., 2021; Baca et al., 2023; Betto et al., 2021; Shi et al., 2021). The rapid evolution of DNA methylation sequencing technology and data analysis strategies holds immense potential to detect causal correlations between specific methylation alterations and particular phenotypes, and to identify precise biomarkers for diagnosis, monitoring disease progression, or assessing treatment efficacy (LaSalle, 2023; Martínez-Iglesias et al., 2023; Ehteshamet al., 2023).

Bisulfite sequencing (BS-seq) is considered the gold standard for achieving base-resolution methylome detection (Dai et al., 2024), as it quantitatively discriminates between unmethylated and methylated cytosines post-bisulfite treatment, as exemplified by whole-genome bisulfite sequencing (WGBS). However, WGBS relies on microgram-level initial input and requires extremely deep sequencing depths, leading to high sequencing expenses. Furthermore, other methodologies inherently suffer from drawbacks in terms of resolution, coverage, sensitivity, and specificity due to limitations in bioinformatics analysis strategies dependent on wildcard or three-letter aligners. These aligners are based on the reduced sequence complexity post-sodium bisulfite treatment, resulting in higher discard rates of unmethylated reads and compromising the accuracy and efficiency of the analyses. Methylation-specific PCR (MSP) provides targeted analysis but is confined to predefined CpG sites, lacking genome-wide applicability. Methylated DNA immunoprecipitation sequencing (MeDIP-seq) allows broad detection of methylated DNA but suffers from low resolution and cannot determine methylation status at individual CpG sites. Methylation-sensitive restriction enzyme sequencing (MRE-seq) captures changes near restriction sites but lacks single-nucleotide resolution. Methylation-sensitive single-nucleotide primer extension (Ms-SNuPE) allows for precise quantification at specific CpG sites, though it is limited by the number of sites that can be assessed simultaneously and requires specialized equipment (Pajares et al., 2021; Beck et al., 2022; Xing et al., 2018; Sun et al., 2018).

**FIGURE 1:**
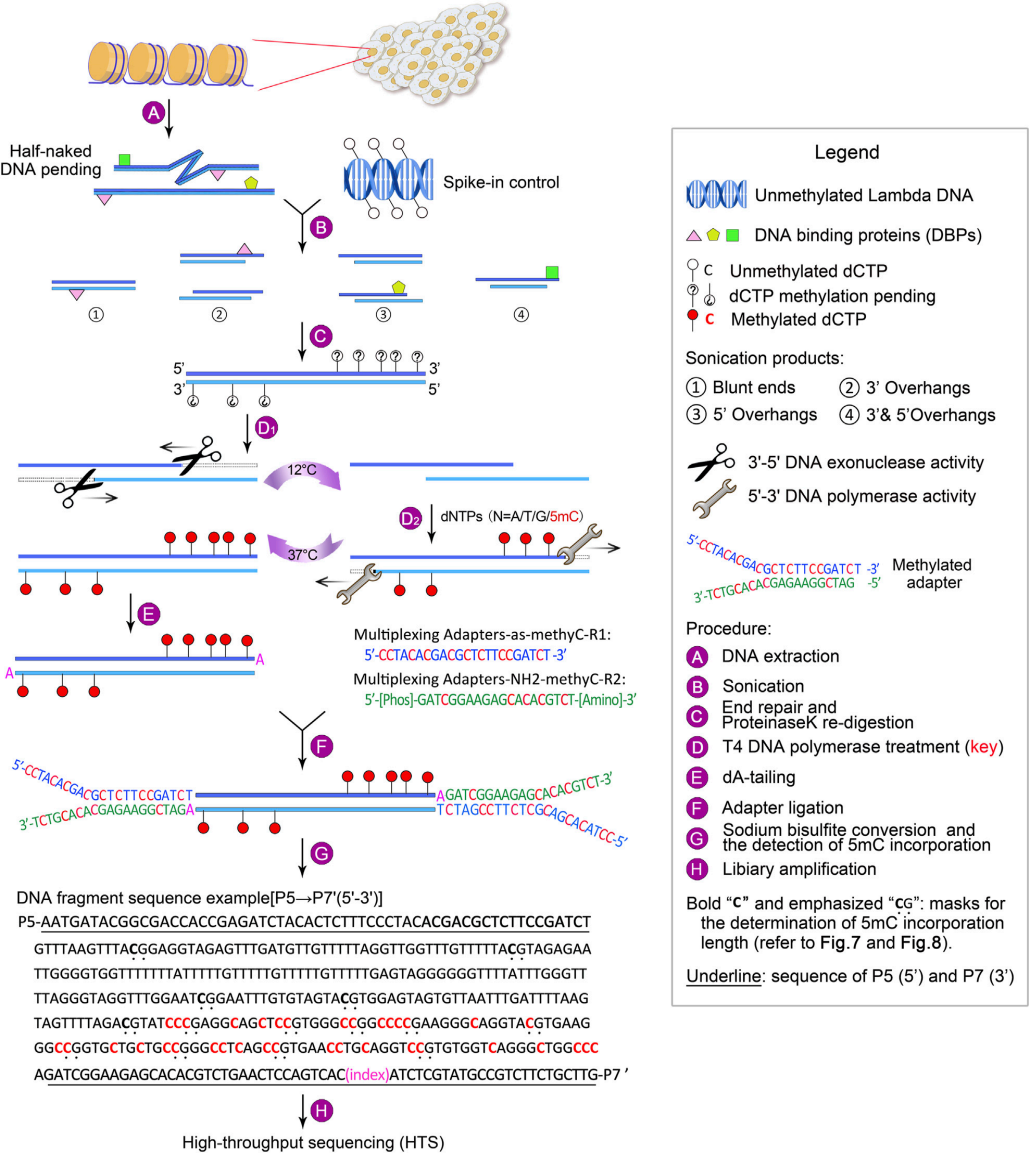
Outline of GPS library construction, ranging from DNA extraction to high-throughput sequencing (HTS). For a detailed description, refer to Experimental design.

**FIGURE 2:**
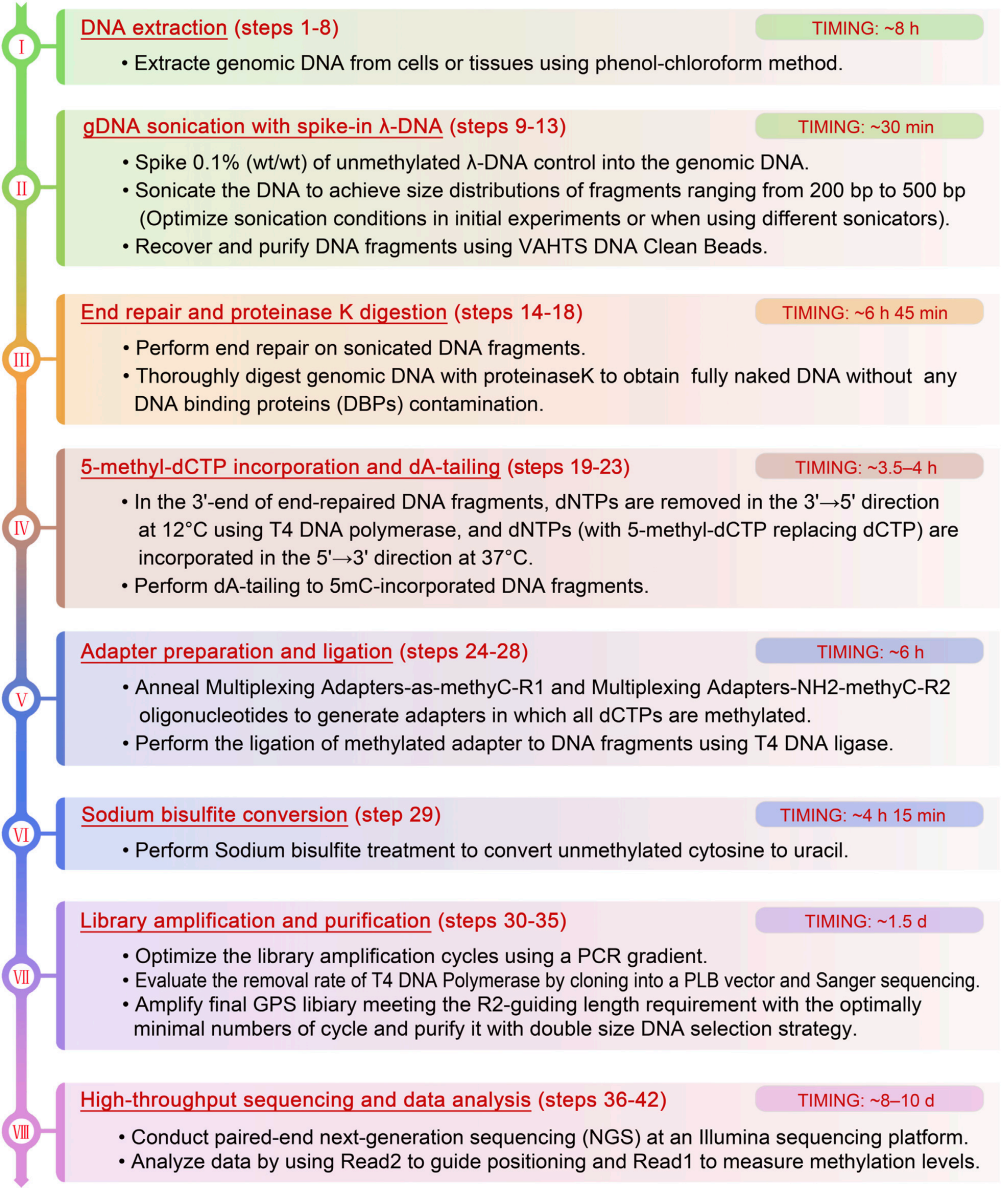
The detailed flowchart of the critical steps and recommendations of GPS library construction.

To overcome the above limitations, we have developed guide positioning sequencing (GPS), a novel library construction method that simultaneously captures DNA methylation and mutation profiles across the entire genome (Li et al., 2019), and assembled a suite of complementary bioinformatics analysis tools. A schematic diagram delineating the GPS protocol is presented in Figure 1. The core principle of GPS lies in the dual functions of T4 DNA polymerase (Speyer and Rosenberg, 1968; Huang and Lehman, 1972; Hershfield and Nossal, 1972), which exhibits exonuclease activity at 12°C, efficiently removing dNTPs from the 3′-end of DNA fragments, while concurrently displaying polymerase activity at 37°C for the incorporation of 5-methyl-dCTP (5 mC). This property preserves cytosines at the 3′-end from bisulfite conversion, retaining the original base composition and facilitating genetic variation profiling at the 3′-end, as well as more rapid alignment of the 5′-end with methylation information to a reference genome. GPS overcomes the limitations of traditional sequencing techniques, particularly excelling in rapid, accurate, and cost-effective diagnosis, treatment, and prognosis of diseases. The comprehensive workflow, encompassing high-throughput sequencing (HTS) and data analysis, can be executed within a timeframe of 15–18 days. All steps are tailored towards achieving the most economical and reliably accurate acquisition of high-quality methylation data (Figure 2).

We have successfully applied the GPS method to generate methylomes of normal and cancer cells from the liver. Remarkably, we have introduced concepts such as methylation of gene body differences to promoters (MeGDP) and methylation boundary shifts (MBS) to elucidate the interplay between methylation and gene expression, which can elucidate changes in cell identity, liver cancer progression, and lung-specific metastasis (Li et al., 2019), and those theories have been widely applied in other research (Wang et al., 2023; Wang et al., 2022b). Furthermore, by integrating GPS into nucleosome occupancy and methylome sequencing (NOME-seq), nucleosome-secured regions (NSRs) have been identified as regions that prevent alterations in cell identity (Luo et al., 2017). In recent research, we utilized DNA probes targeting genes promoter to capture the GPS library for sequencing, thus significantly enriching the reads by two orders of magnitude (BL. Zhang et al., unpublished data). Morever, GPS has been employed across the MCF10 series of cell lines, including MCF-10A, MCF-10AT, MCF-10DCIS, MCF-10CA1h, and MCF-10CA1a, to generate high-quality GPS data for investigating DNA methylation-driven events in the progression and metastasis of breast carcinoma (BRCA). In conjunction with mRNA-seq, we have identified a set of methylation-related differentially expressed genes (mrDEGs) (BL. Zhang et al., unpublished data). Additionally, integrated with bioinformatics tools, such as powerful software for genomic data alignment, methylation site analysis, and pattern recognition, GPS enables more precise analyses and interpretations of complex datasets, leading to the identification of new biomarkers or disease mechanisms. These integrated applications substantially enhance both the research and clinical utility of GPS technology, offering comprehensive genetic and epigenetic insights that aid in the prevention, diagnosis, therapy, and prognosis of diseases.

## 2 Material and equipment

### 2.1 Reagents

▲ CRITICAL: All reagents must be maintained DNase-free and should be prepared in aliquots to mitigate batch contamination.
  - Nuclease-Free Water (not DEPC-Treated) (Invitrogen, cat. no. AM9930)
  - Ethanol (Sinopharm, cat. no. 10009218)
▲ CAUTION: Ethanol is highly flammable.
  - proteinase K (TIAGEM, cat. no. RT403)
  - RNase A (Thermo, cat. no. EN0531)
  - Phenol chloroform (Phenol: chloroform: isoamyl alcohol = 25:24:1, pH > 7.8) (Solarbio, cat. no. P1012-500 mL)
▲ CAUTION: Phenol may cause burns to the skin and eyes. It is imperative to wear protective laboratory clothing, including gloves, a lab coat, and goggles, and to handle phenol within a biological safety cabinet.
  - Agarose (TIANGEN, cat. no. RT101)
  - 100 bp DNA Ladder (TIANGEN, cat. no. MD109)
  - 1 kb DNA Ladder (TIANGEN, cat. no. MD111)
  - 6× loading buffer (TIAGEN, cat. no. RT201)
  - Genegreen Nucleic Acid Dyes (TIAGEN, cat. no. RT210)
  - Ethidium bromide (EtBr, EB)
▲ CAUTION: EtBr is toxic if absorbed through the skin (Yu et al., 2020). To ensure safety, wear PE (polyethylene) gloves under latex gloves when handling this substance.
  - λ-DNA (dam-, dcm-) (Thermo Scientific, cat. no. SD0021)
  - dATP (TaKaRa, cat. no. 4026Q)
  - dGTP (TaKaRa, cat. no. 4027Q)
  - dCTP (TaKaRa, cat. no. 4028Q)
  - dTTP (TaKaRa, cat. no. 4029Q)
  - 5-methyl-dCTP (NEB, cat. no. N0356S)
  - ATP (Thermo Scientific, cat. no. R0441)
  - NEBNext End Repair Enzyme Mix (NEB, cat. no. E6050L)
  - T4 DNA Polymerase (NEB, cat. no. M0203L)
  - Klenow fragment (exo-, 5 U/μL; Thermo Scientific, cat. no. EP0422)
  - T4 DNA ligase (NEB, cat. no. M0202L)
  - Quick ligation kit (NEB, cat. no. M2200L)
  - EZ DNA Methylation-Gold™ Kit (Zymo Research, cat. no. D5006)
  - KAPA HiFi HotStart Uracil + ReadyMix PCR Kit (Kapa Biosystems, cat. no. KK2801)
  - 2× Taq PCR Mix (LifeFeng, cat. no. PT102-02)
  - MinElute Gel Extraction Kit (QIAGEN, cat. no. 28606)
  - DH5α Competent cell (TIANGEN, cat. no. CB101-01)
  - Lethal Based Fast Cloning Kit (TIANGEN, cat. no. VT205-01)
  - VAHTS^®^ DNA Clean Beads (Vazyme, cat. no. N411-02)
  - Qubit dsDNA HS (High Sensitivity) Assay Kit (Life Technologies, cat. no. Q32851)
  - SDS (Amresco, cat. no. S0227-500G)
  - Tris (Sangon Biotec, cat. no. A600194-0500)
  - Acetic acid (Sinopharm Chemical Reagent Co., Ltd. (SCRC), cat. no. 10000218)
  - EDTA (SIGMA-ALDRICH, cat. no. EDS-100G)
  - PBS buffer (pH 7.2; 1X; Gibco, cat. no. 14249-95)

### 2.2 Software

• Any Linux environment
• R project (https://www.r-project.org/)
• NGS QC Toolkit (version 2.3.3) (http://www.nipgr.res.in/ngsqctoolkit.html)
• Trim_galore (version 0.4.0). Freely available at http://www.bioinformatics.babraham.ac.uk/projects/trim_galore/
• FastX toolkit (http://hannonlab.cshl.edu/fastx_toolkit/)
• Bowtie 2 (http://bowtie-bio.sourceforge.net/bowtie2/index.shtml)
• Samtools39. Download from http://samtools.sourceforge.net/
• sw7.0. Download from https://genome.cshlp.org/content/29/2/270/suppl/DC1

### 2.3 Equipment

• Vortex oscillator (Scientific Industries, SI-Vortex-Genie2)
• Manual pipette [Mettler Toledo, cat. no. SL-10XLS (0.5–10 μL); cat. no. SL-100XLS (10–100 μL); cat. no. SL-1000XLS (100–1,000 μL)]
• Tabletop microcentrifuge (VWR MicroStar 17R; cat. no. 521–1647)
• MiniSpin Plus microcentrifuge (Eppendorf, cat. no. 5453000.011)
• Thermal cycler (Biometra TProfessional; cat. no. 846-070-601)
• Erlenmeyer Flask (Zhejiang Mlb Scientific Instruments Co., Ltd., AKM LAB
• Borosilicate Glass Flask Erlenmeyer Flask 250 mL)
• Water bath (Thermo Scientific, cat. no. 2838)
• Sterile Scalpel Blades (Feather, cat. no. 72044)
• Sterile Scalpel Handle (Feather, cat. no. 72040)
• Analytical Balances (METTLER TOLEDO, cat. no. ML204T/00)
• Microwave oven (Samsung MS23F301EAS)
• Gel electrophoresis system (BEIJING LIUYI BIOTECHNOLOGY CO., LTD, cat. no. DYY-10C)
• Electrophoresis cell (Bio-Rad Mini-Sub Cell GT Cell; cat. no. 1704466)
• Gel comb (3 mM; Owl Separation Systems or equivalent)
• Sonicator (Diagenode Bioruptor Twin; cat. no. UCD-400 TO)
• magnetic separation rack (Invitrogen 12321D DynaMag™-2 Magnet)
• Alphaimager UV transilluminator (Alpha Innotech)
• Spectrophotomer (Thermo Scientific NanoDrop 2000/2000C)
• Qubit 2.0 fluorometer (Invitrogen, cat. no. Q32866).
• Qubit assay tubes (Invitrogen, cat. no. Q32856)
• Bioanalyzer 2100 with electrophoresis set (Agilent, cat. nos. G2940CA and G2947CA)
• Illumina Sequencing Platforms (NovaSeq 6000)

### 2.4 Reagents set-up

• Ultrapure Milli-Q-filtered H_2_O. Autoclaved Milli-Q-filtered H_2_O is used throughout the reagent setup.
• Nuclear lysis buffer (50 mM Tris pH 8.1, 10 mM EDTA, 0.3% SDS). This buffer can be stored at RT or 4°C for no more than 1 year.
▲ CRITICAL: White flocculent precipitate will appear when stored at 4°C due to the SDS component. Completely dissolve at 42°C for at least 10 min until the solution turns clear before proceeding to the next step.
   • Unmethylated λ-DNA. To ensure an accurate quantity for the reaction, commercial high-concentration λ-DNA (Thermo Scientific, cat. no. SD0021) should be quantified using a spectrophotometer and then diluted from the original concentration (0.3 μg/μL) to 150 ng/μL, and then to 0.1 ng/μL according to serial dilution.
▲ CRITICAL: The concentration of 150 ng/μL is used for bisulfite enrichment, while 0.1 ng/μL is used for spike-in control. All dilutions should be stored at −20°C for no more than 2 years and must be completely thawed with no visible sediment before use.
• 50× TAE buffer (24.2% Tris, 5.71% acetic acid, 5% EDTA). This buffer can be stored at RT for up to 1 year and should be diluted to 1× before use.
• 1%–2% (wt/vol) agarose gel. Mix and dissolve agarose powder in 1× TAE buffer in an erlenmeyer flask while avoiding foaming [e.g., 1 g of agarose powder in 100 mL of 1× TAE buffer for 1% (wt/vol) or 2 g of agarose powder for 2% (wt/vol)], then heat the solution for 3 min at full power in a microwave with occasional shaking until the agarose has completely dissolved.
▲ CRITICAL: To prevent over-foaming, which leads to a time-consuming dissolving process, microwave the agarose solution in short heating bursts with intermittent shaking. Allow the solution to cool to 50°C for 10 min before pouring it into the gel electrophoresis tank.
▲ CAUTION: Do not remove the Erlenmeyer flask from the microwave immediately after heating to avoid splashing of hot water vapor or boiling substances that can cause burns. Instead, allow the flask to stand in the microwave for at least 5 min, and handle it with insulation gloves and protective clothing.
• Select an appropriate comb size based on the volume of the gel solution to prevent sample overflow and cross-contamination. Carefully pour the gel into the electrophoresis tank, ensuring no bubbles form. The gel typically hardens within about 30 min at RT.
▲ CRITICAL: Remove the comb from the agarose gel slowly and gently to prevent damage to the gel wells. As a precaution, 2 μL of the 6× DNA loading dye can be applied separately to each well to check for leaks.
• The agarose gel can be stained with 100,00× EtBr or EtBr metabolites following agarose gel electrophoresis (AGE).
▲ CAUTION: Ethidium bromide (EtBr) is a potent mutagen that can cause cancer or teratogenic effects. Consequently, the area where EtBr is used must be isolated, and appropriate personal protective equipment such as gloves and masks must be worn. Gloves that have come into contact with EtBr should not be removed from the designated contaminated area.
▲ CRITICAL: The binding of EtBr metabolites, such as GoldView, SYBR Green, or GeneGreen, to DNA can affect its electrophoretic mobility, potentially causing elongated tails or deformed DNA bands and introducing inaccuracies in size estimation. Post-staining of nucleic acids is strongly advocated over pre-staining to mitigate the aforementioned issues during electrophoresis (Konschak and Tinhofer, 2011).
• Methylated adapters. The methylated adapter oligonucleotides, including multiplexing adapters-as-methyC-R1 and multiplexing adapters-NH_2_-methyC-R2 (Supplementary Table S1), in which all dCTPs are substituted by 5-methyl-dCTP, are synthesized using high-performance liquid chromatography (HPLC) for purification. The multiplexing adapters-NH_2_-methyC-R2 requires further modification by adding a phosphoric group to the 5′-end and an amino group to the 3′-end. It is advisable to prepare these adapters in advance; the detailed protocol for synthesizing a 20 μM annealed adapter is outlined in Section 3.7. Divide the annealed adapter solution into 20 μL aliquots and store at −80°C for long-term preservation.
• Primers. All primers are detailed in Supplementary Table S1. Centrifuge the tube at 15,000 × *g* for 3 min, then dilute each primer in 1× TE buffer to a concentration of 10 μM and store at −20°C.
▲ CRITICAL: Slightly open the lid of the tube containing dry primer powder to avoid electrostatic attraction of the powder to gloves.

## 3 Stepwise procedure

### 3.1 DNA extraction — ±8 h

1. Collect cultured cells or pulverize tissues using the liquid nitrogen quick-freezing method, then transfer 460 μL of nuclear lysis buffer into a 1.5 mL Eppendorf tube. Given the high sensitivity of the GPS technique, the volume of nuclear lysis buffer should be adjusted based on the estimated number of cells; for example, 460 μL of buffer can lyse up to 10 million cultured cells or up to 40 mg of tissue. Vigorously vortex the tube until the mixture is homogeneous and viscous.
  ▲ CRITICAL STEP: To preserve the integrity of the DNA content and prevent shearing, avoid using a pipette. To minimize the formation of bubbles, centrifuge at 15,000 × *g* for at least 1 min.
2. Prepare for proteinase K digestion as outlined below. Gently vortex the reaction at RT for at least 1 min. Seal the tube lid with parafilm, centrifuge the tube down briefly, and then incubate at 58°C–65°C for at least 4–6 h to ensure complete digestion of cellular proteins.

 ▲ CRITICAL STEP: Vortex the reaction mixture every 3 h to enhance the digestion process.
3. Return the tube to RT and add 5 μL of RNAse A (10 mg/mL; final concentration 100 μg/mL) to the lysis mixture. Seal the tube lid with parafilm, mix thoroughly by gently vortexing, and then briefly centrifuge to collect the contents. Subsequently, incubate at 37°C for at least 2–3 h to digest cellular RNA.
4. Add an equal volume of 500 μL of phenol-chloroform (phenol: chloroform: isoamyl alcohol at 25:24:1, pH > 7.8) under a fume hood, vortex vigorously for 2 min to mix thoroughly, incubate the mixture at RT for 3 min, and then centrifuge at 15,000 × *g* at 4°C for 15–20 min.
  ▲ CRITICAL STEP: The phenol-chloroform is typically liquid-sealed due to its high volatility. To avoid contaminating the sample with the sealing liquid, use a syringe with a needle to aspirate the lower organic layer, then discard the needle and transfer the clean phenol-chloroform solution to a new sterile Eppendorf tube. Additionally, the low viscosity of phenol-chloroform can cause difficulties in accurate aspiration and potential leakage from the tube lid. These issues can be mitigated by lubricating the inner wall of the pipette tip and securely fastening the lid hinge during vortexing.
  ▲ CAUTION: Phenol-chloroform is toxic; handle it under a fume hood to prevent poisoning.
5. Aspirate 400 μL of the supernatant from the upper aqueous layer and transfer it to a new microcentrifuge tube using a 1,000 μL pipette tip. To precipitate DNA, combine the reagents as follows. Vigorously vortex the reaction mixture and incubate on ice for 5 min to promote DNA precipitation. Centrifuge at 15,000 × *g* for 20–30 min at 4°C to collect the DNA as a white pellet.
  ▲ CRITICAL STEP: After centrifugation, visible layer stratification occurs. Modify a 1,000 μL pipette tip by cutting it with a razor to widen its diameter and carefully aspirate the supernatant, avoiding contact with the middle or lower layer, as contamination from these layers can significantly impair subsequent enzymatic reactions. If proteins or organic solvents are accidentally aspirated, add 100 μL of H_2_O to achieve a total volume of 500 μL, and then repeat step 4. Place the tube in the centrifuge with the lid hinge facing outward to facilitate the identification and handling of the DNA pellet during rinsing.
6. Aspirate the supernatant using a 1,000 μL pipette and discard it. Add 900 μL of 75% (vol/vol) ethanol to the tube, and invert it 6–8 times. Centrifuge at 15,000 × *g* at 4°C for 5–10 min. Repeat this washing step once more.
7. Aspirate and discard the supernatant. To collect any remaining liquid droplets on the inner walls of the tube, centrifuge briefly at 15,000 × *g* at RT for 10 s, then carefully remove the residual liquid with a 20 μL pipette tip.
8. Allow the pellet to air dry at RT for 2–5 min to ensure all traces of ethanol are removed. Elute the gDNA with 50 μL of DNA elution buffer [10 mM Tris-HCl (pH 9.0) and 0.1 mM EDTA] or nuclease-free water. Measure the concentration using a spectrophotometer with 1 μL of the solution.
  ▲ CRITICAL STEP: Add the DNA elution buffer when the periphery of the pellet turns transparent. Avoid over-drying the pellet, as this can make it difficult to dissolve in the buffer. To assess DNA purity and concentration, measure the absorbance ratios at 260/280 nm and 260/230 nm using a spectrophotometer. DNA samples with protein and/or RNA contamination should not be used for GPS.
  ■ PAUSE POINT: Purified gDNA can be stored at −20°C for long-term storage.

**Table:** 

Component	Volume (μL)	Final concentration
ProteinaseK (20 mg/mL)	20	0.05–1 mg/mL
SDS (10%)	20	0.4%
Nuclear lysis buffer	460	
Total	500	

**Table:** 

Component	Volume (μL)
Isopropanol	400
3 M Sodium acetate (pH 5.2)	40
Aqueous (top) phase	400
Total	840

### 3.2 gDNA sonication with spike-in λ-DNA — ±30 min

**FIGURE 3:**
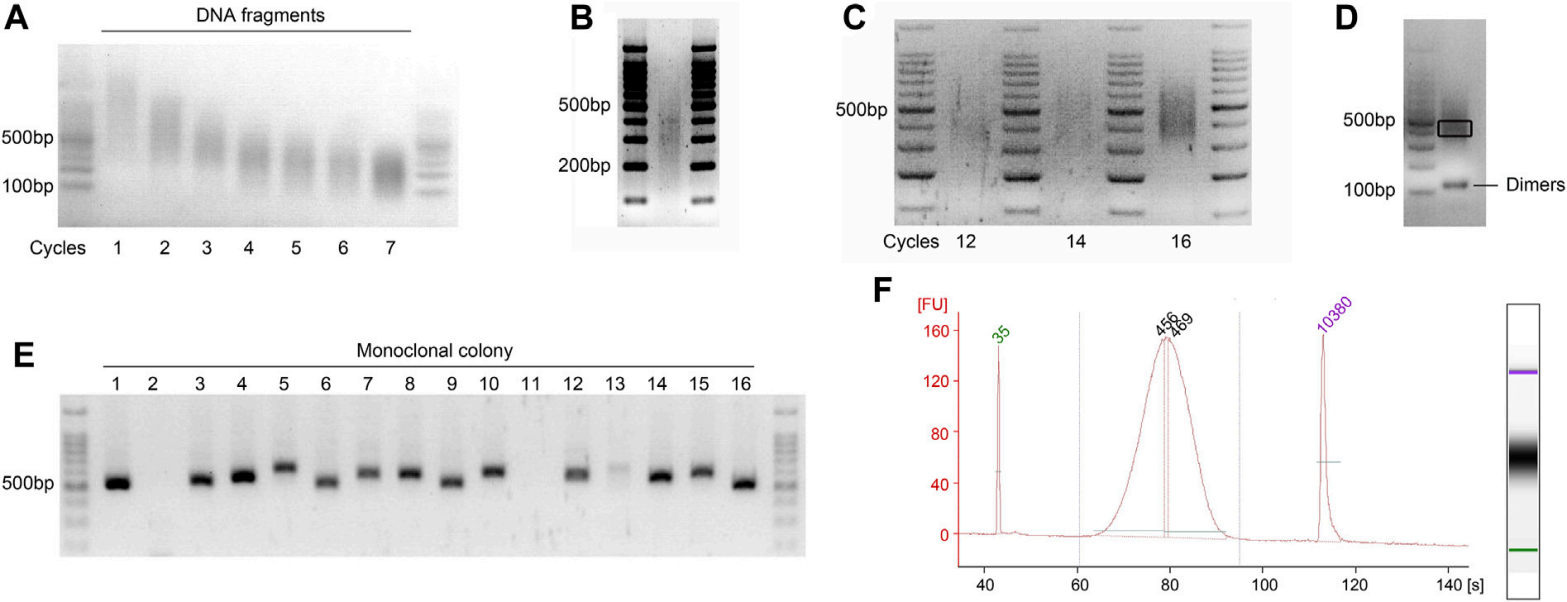
Visualization of the quality check steps during GPS library preparation. Agarose gels viewed under a UV transilluminator. The most intense band in the 6 μL DNA ladder (100 bp ladder) is 500 bp of in size, containing 100 ng of DNA. **(A)** Representative gel image showing optimization of the sonication cycling conditions for input DNA. Run on a 2% (wt/vol) agarose gel to determine the optimal number of sonication cycles for obtaining fragments between 200 and 500 bp. The illustration showing the optimal cycle of 4. **(B)** Optimal distribution of sonicated fragments exhibits maximum enrichment at 300–350 bp. **(C)** Determination of the optimal cycle number for final library amplification. Pre-amplifications using 1/20 volumes (vol/vol) of bisulfite-converted DNA were performed to ascertain the ideal cycle number. The results demonstrate that cycle numbers of 12 and 14 are insufficient, while 16 is excessive; thus, the optimal number is 15. **(D)** Final library of GPS was run on 2% (wt/vol) agarose gel, demonstrating an enrichment at approximately 450 bp. A gel slice, highlighted in black frame and corresponding to fragments ranging from 400 to 500 bp, was excised with a sterile scalpel to inspect the incorporation length of 5 mC. Additionally, visualization of potential primer dimer contamination that may occur during the library PCR amplification was conducted. **(E)** Colony PCR is performed to screen for at least 10 positive clones with a band size of 520–620 bp, as shown for the monoclonal colony 1, 3–10, and 12–16. **(F)** Electropherograms of High-Sensitivity chips of the final GPS library on an Agilent Bioanalyzer 2100. The PCR amplification primers, which are 122 bp and meet the requirements of the Illumina platform, are connected to both ends of the fragment; accordingly, the peak increased to ∼450 bp. FU, fluorescence units.

1. Spike 0.1% (wt/wt) unmethylated λ-DNA control into the gDNA of interest (e.g., 0.1 ng of λ-DNA for 100 ng DNA). Set up the DNA sonication reaction according to the following scheme:
  ▲ CRITICAL STEP: For an adequate yield, it is recommended to start with an initial DNA quantity of 1 μg.
2. Prechill the sonicator water bath by adding ice 15 min in advance.
  ▲ CRITICAL STEP: Maintain the DNA temperature below 10°C during and after sonication. Distribute a thin layer of ice on the surface of the water, as a thick ice layer can hinder ultrasound penetration. Alternatively, consider using a more advanced ultrasonicator equipped with circulating ice-cold water. If the sonicator is operated continuously for 1 hour, it must be cooled for 30 min to maintain the ultrasound’s strength and stability.
3. Distribute a thin layer of ice on the water’s surface and place the microcentrifuge tube containing DNA in the tube holder, ensuring the holder is balanced.
  ▲ CRITICAL STEP: Ensure the sonicator holder remains balanced to guarantee uniform ultrasound intensity across all tubes.
4. Sonicate the DNA to achieve the desired range of sheared fragment sizes (200–500 bp). For this process, we utilized a Qsonica Q700 sonicator set to 80% power amplitude, with 3–4 cycles, each consisting of 2 sets of 30-s ON and 30-s OFF pulses.
  ▲ CRITICAL STEP: Briefly vortex and centrifuge the DNA samples between each cycle. Post-sonication, keep the sheared DNA fragments on ice to enhance the efficiency of subsequent end repair. The precise sonication parameters should be optimized prior to sample preparation due to variations among different sonicator models and AGE setups (Figure 3A).
  ▲ CAUTION: Always wear ear protection when operating the sonicator.
  ■ PAUSE POINT: The sonicated gDNA can be stored at −20°C for up to 1 year. It is strongly recommended to proceed immediately to the next step.
5. Assess the efficiency of sonication by analyzing 1 μL of sheared gDNA using an Agilent Bioanalyzer 2100 to inspect the size distribution of the DNA fragments. Alternatively, mix 5 μL of sheared gDNA with 1 μL of 6× DNA loading buffer and run on a 2% (wt/vol) agarose gel at 150 V for 30 min after a brief centrifugation. A smear should appear between 200 and 500 bp, with the most intense enrichment around 300–350 bp (Figure 3B).
  ▲ CRITICAL STEP: To prevent cross-contamination resulting from samples floating out of the agarose wells, it is advisable to use 6× DNA loading buffer containing glycerin and bromophenol blue. Ensure that the ultrasound-fragmented DNA is neither too short nor too long. Excessively short fragments may be lost during subsequent digestion steps, while overly long fragments can compromise the diversity of data outputs during sequencing, adversely affecting the quality of the library.

**Table:** 

Component	Volume (μL)	Final concentration
Purified DNA	100–500 ng	
Lambda-DNA (0.1 ng/μL)	1–5 μL	0.1%
dd H_2_O	Variable	
Total	85 μL	

### 3.3 End repair of DNA fragments — ±1 h 45 min

1. Proceed to set up the end repair reaction in a sterile 1.5 mL Eppendorf tube on ice as follows. Ensure that the buffer is completely thawed without any visible sediment.

2. Centrifuge the tube down at 15,000 × *g* for 10 s and, then incubate in an Eppendorf Thermomixer at 20°C for 1 h.
3. Place the reaction on ice after incubation. Centrifuge at 15,000 × *g* for 10 s, purify the DNA sample using equal volumes (100 μL) of VAHTS DNA Clean Beads (BOX 1) and elute in 42.5 μL of nuclease-free water.
  ▲ CRITICAL STEP: Prepare a master mix and dispense aliquots into each reaction tube when processing multiple samples. The stabilizing effect of low temperatures on hydrogen bonds contributes to a lower probability of base pair opening. During end repair, maintaining a low temperature is crucial to prevent the disruption of new hydrogen bonds by increasing temperature, which can hinder the efficient formation of 3′–5′ phosphodiester bonds (Chen and Prohofsky, 1996; Osman et al., 2021).
  ■ PAUSE POINT: End-repaired DNA can be stored at −20°C for at least 6 months or at −80°C for long-term storage.

BOX 1 **Efficient purification to minimize DNA loss — ±30–40 min.**

Procedure

▲CRITICAL: DNA recovery after steps of reactions is essential but inevitably leads to a loss of DNA. To minimize interference from ethanol and salt residues in subsequent enzymatic reactions, DNA purification magnetic beads are preferred over columns, particularly for low input DNA. Significant loss of beads can directly impact the quantity of purified DNA. The steps and tips provided below ensure maximum efficiency in purification and minimal loss of beads. The recovery ratios are summarized in the Anticipated Results section. The entire process is conducted at RT.

Prepare magnetic beads and fresh 80% alcohol in advance

 1. Take out the VAHTS DNA Clean Beads from the fridge and flick the tube, gently pipette the solution up and down, or slowly vortex the tube to resuspend the beads. Ensure the beads are thoroughly mixed with the buffer before adding them to the sample. Let the tubes warm up at RT for at least 10 min.
 2. Prepare fresh 80% ethanol wash solution with nuclease-free water.
  ▲CRITICAL STEP: It is strongly recommended to use the fresh alcohol. Test the flammability of freshly prepared alcohol in a biological safety cabinet to confirm the concentration is 80%, not 20%, which is a crucial and often overlooked step.

Purification process and tips

3. Accurately measure the DNA volume and add an equal volume of VAHTS DNA Clean Beads (1:1 vol/vol) to the solution containing DNA.
4. Mix completely and gently by pipetting up and down at least 20 times with a 200 μL pipette tip, avoiding bubble formation.
5. Allow the mixed sample to sit at RT for 5 min to facilitate bead-DNA complex formation.
6. Place the tube on the magnetic separation rack for at least 5 min until the solution turns clear.
7. Slowly aspirate and discard the supernatant, leaving about 2 μL in the tube to avoid disturbing the beads.
8. Add 200 μL of 80% (vol/vol) ethanol into the tube from the opposite side of the magnetic beads. Rotate the tube clockwise or counterclockwise on the magnetic separation rack to facilitate washing and reassemble the fallen magnetic beads, and then incubate at RT for 5 min again, followed by discarding all the supernatant each time. Repeat the washing step once more.
9. Remove the tube from the magnetic separation rack and centrifuge at ×4,000 *g* for 10 s to collect remaining liquid on the inner wall of the tube, then quickly place the tube back on the magnetic rack for adsorption.
10. Carefully remove residual ethanol with a 20 μL pipette tip and let the beads air dry with the lid open for 10 s to 1 min, depending on the bead quantity. Ensure all ethanol has evaporated and no cracks appear on the surface of the magnetic beads.
   ◆ TROUBLESHOOTING
11. Remove the tube from the magnetic rack and elute the dried bead pellet in 42.5 μL of nuclease-free water.
12. Vortex gently for several seconds, then let sit at RT for 5 min.
13. Centrifuge at 15,000 × *g* for 1 min to encourage bead aggregation, then quickly place the tube back on the magnetic separation rack for 5 min.
14. Transfer 41 μL of the DNA solution to a new 1.5 mL Eppendorf tube without disturbing the beads, and measure the DNA concentration using a Nanodrop 2000 or Qubit 2.0 fluorometer with 1 μL of the solution.
   ▲CRITICAL STEP: Ensure that all ethanol is removed, as the 5 mC incorporation step is sensitive to traces of buffer and/or ethanol. Avoid over-drying the beads, as they may become flaky and crack, reducing DNA recovery efficiency. Make sure the lid hinge faces outward during each centrifugation step to facilitate handling.
   ◆ TROUBLESHOOTING

### 3.4 Proteinase K digestion and purification — ±5 h

1. Perform the digestion at 58°C–65°C overnight or for at least 4–6 h to obtain fully naked DNA. The digestion reaction includes the reagents as described below:

 ▲ CRITICAL STEP: proteinase K digestion is a critical step for obtaining completely naked DNA by digesting all DBPs.
2. Purify the DNA sample using equal volumes (50 μL) of VAHTS DNA Clean Beads (BOX 1), elute in 52.5 μL of nuclease-free water, and measure the concentration with 1 μL of solution using a spectrophotometer.

**Table:** 

Reagent	Volume (μL)
Fragmented DNA	80
NEBNext end repair reaction buffer (10×)	10
NEBNext end repair enzyme mix	5
Nuclease-free water	5
Total	100

**Table:** 

Component	Volume (μL)	Final concentration
Proteinase K (20 mg/mL)	2	0.05–1 mg/mL
SDS (10%)	2	0.4%
End-repaired DNA fragements	40	
Nuclease-free water	6	
Total	50	

### 3.5 Substitute dCTP with 5-methyl-dCTP at the termini of end-repaired DNA fragments — ±2–2.5 h

▲ CRITICAL: The catalytic functions of T4 DNA polymerase enable the removal of dNTPs at the 3′-end in a 3′→5′ exonuclease direction at 12°C in the absence of dNTPs, and facilitate the incorporation of dNTPs in a 5′→3′ polymerization direction at 37°C in the presence of dNTPs, where 5-methyl-dCTP replaces dCTP. All procedural steps are performed on ice, and all reagents are added to the bottom of the tube using a vertically held pipette. Mixing is accomplished with a pipette tip to ensure no reagent residue remains on the inner wall of the tube.

#### 3.5.1 Removal of dNTPs in the 3′→5′ direction — ±1–1.5 h

1. Dilute four commercial dNTPs (100 μM; dATP, dTTP, dGTP, and 5-methyl-dCTP) to 10 μM. Then, in a sterile 1.5 mL Eppendorf tube, vigorously mix an equal volume of each dNTP to achieve a final concentration of 2.5 μM for each dNTP.
2. Set up the digestion reaction in a sterile 0.2 mL Eppendorf tube according to the scheme outlined below:

3. Incubate the tube in a thermocycler set to 12°C for an optimized duration based on the digestion rate of T4 DNA polymerase to excise 36–150 dNTPs. Ensure the heated lid is turned off or maintained at 20°C to prevent enzyme deactivation due to heat radiation from prematurely elevated temperatures.
  ▲ CRITICAL STEP: The removal of dNTPs is pivotal for GPS, necessitating precise control over the digestion time of T4 DNA polymerase. All steps must be conducted on ice, and the thermocycler should be pre-cooled to 12 °C. T4 DNA polymerase should be added last to the reaction mixture and immediately placed into the pre-cooled thermocycler. Divide commercial T4 DNA polymerase into 3.25 μL aliquots in sterile 0.2 mL Eppendorf tubes and store at −20 °C to ensure accurate quantification and to prevent activity loss from repeated freeze-thaw cycles. Note that the activity can vary between different batches of T4 DNA polymerase. For new enzyme batches, it is strongly recommended to perform a time gradient test to determine the optimal digestion speed (1, 1.5 and 2 h). If the initial DNA input is less than 500 ng, reduce the enzyme quantity and adjust the digestion conditions correspondingly.
  ◆ TROUBLESHOOTING

#### 3.5.2 Incorporation of dNTPs in the 5′→3′ direction and inactivation of T4 DNA polymerase — ±1 h

1. Add 2.5 μL 5 mC-containing dNTPs and 0.3 μL NEB Buffer 2 (10×) to the tube post-incubation. Mix thoroughly using a 200 μL pipette tip, then place the tube in a thermocycler set to 37 °C with the lid temperature at 42 °C for 30 min.
2. Inactivate T4 DNA polymerase by raising the thermocycler temperature to 75 °C and the lid temperature to 85 °C for 20 min.
  ▲ CRITICAL STEP: Once dNTP removal is complete, immediately add 5 mC-containing dNTPs to the reaction system on ice. To circumvent the thermal radiation effect where the heated lid temperature could impede T4 enzyme efficiency, it is highly advisable to use three separate thermocyclers preset to the respective temperatures. Transfer tubes swiftly to avoid delays that could lead to enzyme instability.

### 3.6 dA-tailing reaction — ±1.5 h

1. Add NEB Buffer 2 (10×), Klenow fragment (3′→5′ exo-), and dATP (10 mM) to the tube containing 5 mC-incorporated DNA on ice according to the following scheme:

2. Incubate the tube in an Eppendorf Thermomixer set at 37 °C with a lid temperature of 42 °C for 1 h.
3. Transfer the entire reaction volume (56.1 μL) to a sterile 1.5 mL Eppendorf tube and place on ice immediately after incubation.
4. Purify the DNA sample using an equal volume (56.1 μL) of VAHTS DNA Clean Beads (BOX 1). Elute the DNA in 17.5 μL of nuclease-free water and measure the concentration using 1 μL of the solution with a spectrophotometer. Note that fragments shorter than 300 bp may be digested due to the removal of 36–150 dNTPs from both 3′-end by T4 DNA Polymerase, resulting in a relatively low DNA recovery rate (approximately 50%–60%).
  ▲ CRITICAL STEP: Once the dA tailing is complete, the samples must be placed on ice immediately.
  ■ PAUSE POINT: dA-tailed purified DNA can be stored at −20 °C for up to 6 months.

**Table:** 

Reagent	Volume/mass
NEB Buffer2 (10x)	5 μL
Naked DNA fragments	500 ng
T4 DNA Polymerase	3.25 μL
Nuclease-free water	Variable
Total	50 μL

**Table:** 

Reagent	Volume (μL)
5 mC-incorporated DNA	52.8
NEB Buffer 2 (10×)	0.3
Klenow Fragment (3′→5′ exo-)	1.5
dATP (10 mM)	1.5
Total	56.1

### 3.7 Adapter preparation and ligation — ±6 h

1. Anneal the multiplexing adapters-as-methyC-R1 and multiplexing adapters-NH_2_-methyC-R2 oligonucleotides to produce a 20 μM adapter. Dilute the oligonucleotides to 100 μM and mix the annealing reaction components listed in the following table in a sterile 1.5 mL Eppendorf tube on ice. Ensure all reagents are completely thawed without any visible sediment. Details regarding the oligonucleotides’ sequences and synthesis are provided in Supplementary Table S1 and the Reagent Setup section.

2. Distribute 100 μL of anneal system into two sterile 0.2 mL Eppendorf tubes (50 μL each) and conduct the reaction in a preheated thermocycler using the specified program. Alternatively, incubating and cooling the sample in a water bath within a 500 mL beaker from 98°C to RT in a sterile 1.5 mL Eppendorf tube sealed with parafilm is also a simple and effective method. The annealing program is as follows: 90 °C for 1 min; 80 °C for 1 min; 70 °C for 1 min; 60 °C for 1 min; 50°C for 10 min; 48°C for 10 min; 45°C for 15 min; 42°C for 20 min; 40°C for 20 min; 38°C for 20 min; 35°C for 15 min; 32°C for 10 min; and 30°C for 5 min.
  ▲ CRITICAL STEP: To prevent sample evaporation, utilize a heated thermocycler lid set at 95°C. If employing a water bath for incubation, centrifuge the tube after 30 min and return it quickly to the bath. To prevent self-ligation of the multiplexing adapters-as-methyC-R1 and the formation of primer dimers during library amplification (Figure 3D), add multiplexing adapters-as-methyC-R1 on ice and anneal immediately.
3. Prepare the ligation reaction on ice using a sterile 0.2 mL Eppendorf tube, adding the components listed in the following scheme. Ensure that all reagents are completely thawed without any visible sediment before use. Alternatively, conducting the ligation reaction overnight (6–8 h) with standard T4 DNA Ligase (M0202S; NEB) significantly enhances ligation efficiency.

 ▲ CRITICAL STEP: Keep all components on ice and mix immediately after adding Quick ligase. We recommend supplementing with additional ATP, a crucial cofactor that catalyzes the formation of phosphodiester bonds by DNA ligase (Shi et al., 2018; Lei and Hili, 2017). To prevent ATP degradation, minimize repeated freeze-thaw cycles. Place the tubes in a thermocycler set to 18°C for 2 h, then incubate at 4°C with the thermocycler lid open. An additional 30 min of incubation at RT may enhance ligation efficiency.
4. Purify the post-ligation DNA sample with equal volumes (25 μL) of VAHTS DNA Clean Beads (BOX 1).
  ▲ CRITICAL STEP: VAHTS DNA Clean Beads are effective in removing excess adapters and preventing the self-ligation of multiplexing adapters-as-methyC-R1, crucial for reducing primer dimers that interfere with PCR library amplification efficiency. These dimers can also be eliminated by employing a double size DNA selection strategy.
5. Elute the purified DNA in 16.5 μL of nuclease-free water and measure the concentration using 1 μL of the solution with a spectrophotometer. The total DNA amount may increase due to the adapter ligation at both ends of the DNA fragments. The remaining samples will undergo bisulfite conversion as outlined below.
  ■ PAUSE POINT: The post-ligation sample can be stored in elution buffer at −20°C for up to 6 months.

**Table:** 

Reagent	Volume (μL)
Multiplexing Adaptors-as-methyC-R1 (100 μM)	20
Multiplexing Adaptors-NH_2_-methyC-R2 (100 μM)	20
NEBT4 DNA Ligase Reaction Buffer (10×)	10
ATP (10 mM)	10
Nuclease-free water	40
Total	100

**Table:** 

Reagent	Volume (μL)
Quick ligase reaction buffer (2×)	20
ATP (25 mM)	1.5
Quick ligase	2
Methylated Adaptor (20 uM)	1
dA-tailed DNA from Step 23	15.5
Total	40

### 3.8 Sodium bisulfite conversion of adapter-attached DNA frangments — ±4 h 15 min

**FIGURE 4:**
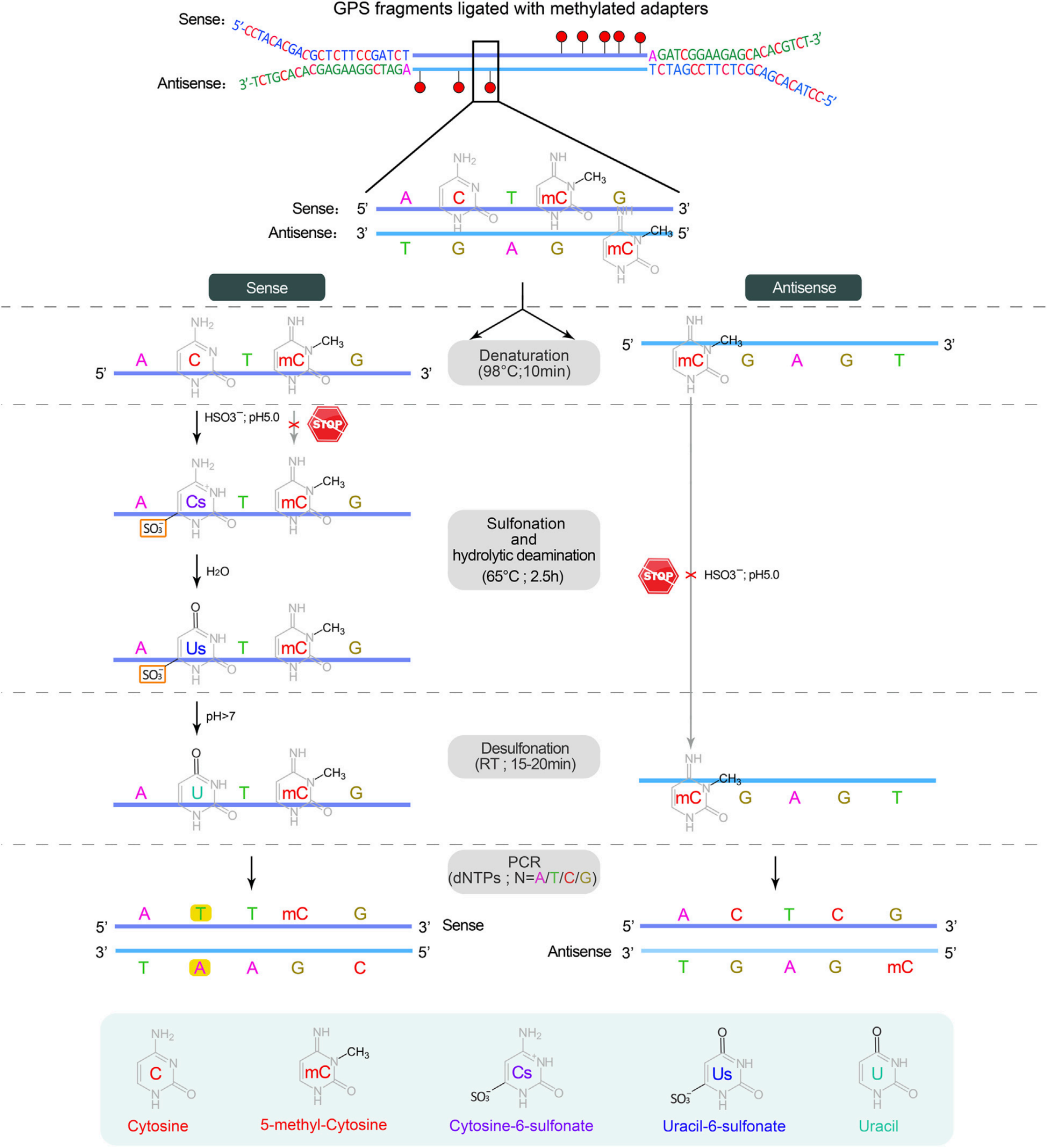
The sodium bisulfite conversion and PCR amplification. Bisulfite treatment is carried out on a single strand DNA derived from the denaturation of double-stranded DNA. The first two steps, sulfonation and hydrolytic deamination, are performed in a thermocycler, while the desulfonation occurs anywhere at room temperature (RT, 20°C–30°C), resulting in the sequential conversion of unmethylated cytosine into cytosine-6-sulfonate, uracil-6-sulfonate, and finally uracil, while methylated cytosine remains unchanged. In subsequent PCR cycles, uracil on both strands is substituted by thymine, completing the conversion from cytosine to thymine.

▲ CRITICAL: Sodium bisulfite conversion of cytosine to uracil involves three independent chemical reactions: sulfonation, hydrolytic deamination, and desulfonation. The methyl group on cytosine at C5 prevents sulfonation by inhibiting nucleophilic attack at C6 due to its electron-donating properties, which fails to convert cytosine to uracil, serving as a means to distinguish between methylated and unmethylated cytosines (Wang et al., 1980; Vaisvila et al., 2021) (Figure 4).

#### 3.8.1 Preparation of sodium bisulfite mix — ±15 min

1. Dissolve the CT conversion reagent (10 reactions per tube) according to the EZ DNA Methylation-Gold Kit instructions. Vortex and incubate at 37°C while shaking at 1400 rpm using an Eppendorf Thermomixer for at least 10 min until all precipitates are completely dissolved. Aliquot 130 μL of the reagent into sterile 0.2 mL Eppendorf tubes. The components are as described in the following table:

 ▲ CRITICAL STEP: The fully dissolved bisulfite CT conversion reagent should be used immediately for analysis or can be stored protected from light at 4°C for up to 1 week or at −20°C for 1 month. It is recommended to aliquot 130 μL into sterile 0.2 mL Eppendorf tubes, clearly labeled with the date and volume, to avoid repeated freeze-thaw cycles. The frozen bisulfite CT conversion reagent must be completely thawed and thoroughly mixed without any visible sediment by incubating at 37°C; avoid placing it on ice prior to use as it may compromise the reagent’s efficiency.

**Table:** 

Component	Volume/mass
M-Dissolving Buffer	50 μL
M-Dilution Buffer	300 μL
CT Conversion Reagent	1 tube
Nuclease-free water	900 μL

#### 3.8.2 Sulfonation and hydrolytic deamination in a thermocycler — ±3 h

Add 15 µL of adapter-ligated DNA and 5 µL of unmethylated λ-DNA (150 ng/μL) to 130 µL of CT conversion reagent, bringing the total volume to 150 µL. Mix thoroughly and place in a thermocycler to perform the program outlined below:

 ▲ CRITICAL STEP: Adjust the solution volume according to the maximum capacity of the thermocycler model; alternatively, the volume can be divided into multiple tubes. Adding λ-DNA to each sample before bisulfite conversion not only prevents loss of gDNA and increases the recovery yield but also provides an adequate amount of substrate for chemical conversion, which enhances the reproducibility and robustness of the bisulfite conversion reaction.
  ■ PAUSE POINT: The solution can be stored at 4°C for no more than 20 h after sulfonation and hydrolytic deamination. Immediate desulfonation is recommended.

**Table:** 

Step	Temperature (°C)	Time
Denaturation	95	10 min
Incubation	65	150 min
Hold	4	Indefinite

#### 3.8.3 Desulfonation at RT — ±1 h

1. Add 600 µL of M-Binding Buffer to a Zymo-Spin™ IC Column.
2. Transfer all 150 μL of the solution into the IC Column and close the lid. Mix gently by inverting the column 6–8 times and allow the column to stand for 2 min at RT. Centrifuge at 3,000 × *g* for 1 min, then at 15,000 × *g* for 30 s, and discard the flow-through.
  ▲ CRITICAL STEP: Low-speed centrifugation during this step may promote DNA adsorption onto the column.
3. Pipette 100 µL of M-Wash Buffer into the column, and incubate for 2 min at RT. Centrifuge at 15,000 × *g* for 1 min and discard the flow-through. Transfer the spin column back into a new sterile 2 mL collection tube.
4. Add 200 µL of M-Desulphonation Buffer to the column, immediately close the column lid, and incubate at RT for 20 min. Then, centrifuge at 15,000 × *g* for 30 s.
  ▲ CRITICAL STEP: Tightly close the column lid to protect the reaction from acidification due to carbon dioxide in the air, which maintains a pH > 7 for an efficient desulfonation reaction.
5. Pipette 200 µL of M-Wash Buffer into the column and let it stand for 2 min. Centrifuge at 15,000 × *g* for 30 s and discard the flow-through Repeat this step one more time.
6. Centrifuge in a new sterile 2 mL collection tube for 1 min at 15,000 × *g.*
7. Transfer the IC Column into a sterile 1.5 mL Eppendorf tube, and leave it at RT for 2 min with the lids open to completely dry the membrane of the column.
8. Carefully pipette 11.5 μL of preheated nuclease-free water (or Buffer EB) directly onto the center of the membrane and incubate at RT for 3 min with the lid closed. Centrifuge for 2 min at 15,000 × *g* to collect the bisulfite-treated DNA. Repeat this step to achieve a total volume of 20 μL.
  ▲ CRITICAL STEP: Elute the DNA twice with preheated nuclease-free water at 50°C to improve DNA yield. It is strongly recommended to perform test library amplification immediately and store the remaining solution at −80°C after bisulfite treatment.
  ■ PAUSE POINT: The bisulfite-converted dsDNA is relatively unstable and should be temporarily stored at −80°C for no longer than 3 months.
  ◆ TROUBLESHOOTING

### 3.9 Library amplification — ±1.5 days

 ▲ CRITICAL: To construct a qualified library, it is necessary to perform test library amplification to determine the optimal cycle number and Sanger sequencing to verify the removal length of 5 mC incorporation.

1. Perform library pre-amplification with 1 μL [1/20 volumes (vol/vol) of bisulfite-converted DNA] of template to optimize the number of cycles needed. Thaw the 2× KAPA HiFi HotStart Uracil + ReadyMix, PCR.primer.PE.1.0 (10 μM), and Multiplexing PCR Primer-Index (10 μM) and mix all components in a sterile 0.2 mL Eppendorf tube according to the following scheme. It is recommended to perform under a clean laminar flow hood.

 ▲ CRITICAL STEP: The KAPA HiFi DNA polymerase, whose uracil-binding pocket has been inactivated, is strongly recommended for the amplification of bisulfite-converted DNA template because the uracil-binding pocket of traditional DNA polymerase in nature binds uracil base prevents the accumulation of mutations caused by cytosine to uracil (Ahn et al., 2019).
2. Place the PCR reaction tube in a preheated thermocycler according to the program as described below. Set 12, 14, and 16 cycles for three different reactions to determine the optimal cycle number by AGE. Once the optimal cycle number is determined, can 8 μL of template be used to amplify the final library only if the removal length of the 3′-end has been verified. The remaining DNA volume (9 μL) can be used as a backup.

 ▲ CRITICAL STEP: We recommend performing all pre-PCR steps under a clean laminar flow hood and use aerosol-resistant pipette tips and sterile tubes/plasticware to prevent cross-contamination of different libraries and other sources. The optimal cycle number for each library must be determined separately by AGE when constructing multiple libraries at the same time to ensure high sequence diversity and low duplicate rates.
 ▲ CRITICAL STEP: For the construction of the final library, determine the minimum number of PCR cycles required based on the quantity of the template DNA. Decrease the number of PCR cycles by one each time the amount of DNA template is doubled (e.g., use 15 cycles for 1 μL of template and 12 cycles for 8 μL of template).
3. Electrophorese 30 μL of the library mixed with 6× DNA loading dye on a 2% agarose gel at 150 V for 40–45 min. Load 6 μL of a 100 bp DNA ladder in the first and last lanes as marker. Stop the electrophoresis when the bromophenol blue dye nearly reaches the bottom of the gel.
   ▲ CRITICAL STEP: Use a comb with larger wells to fully accommodate 25–30 μL of the sample when preparing a 2% agarose gel. Leave at least one empty lane between samples if processing multiple samples to avoid cross-contamination.
4. Visualize the gel under a UV transilluminator. The library should display a size range of 400–500 bp with a peak around 450 bp. Determine the optimal number of PCR cycles based on the intensity of the band. The recommended quantity of the purified library should be at least 40 ng (Figure 3C).
  ▲ CRITICAL STEP: Place a piece of plastic wrap on the UV lamp platform to prevent cross-contamination during gel cutting. For enhanced band visibility, cool the gel for several min before inspection.
  ▲ CAUTION: Wear protective clothing and a face shield to avoid exposure to carcinogenic UV light.
  ◆ TROUBLESHOOTING
5. Identify the removal rate of dNTPs by T4 DNA polymerase as described in BOX 2.

BOX 2 **Determining the removal rate of dNTPs by T4 DNA polymerase — ±1.5 days.**

Procedure

▲ CRITICAL: The length of DNA excised by T4 DNA polymerase should be 36–150 bp, which is crucial for paired-end sequencing and subsequent data analysis. The pre-amplification GPS library can be retrieved, cloned into the PLB vector, and sequenced to estimate the distribution of exonuclease activity based on the last cytosine at the 3′-end or the first guanine at the 5′-end.

Agarose gel cutting and recovery of 400–500 bp amplified library fragments — ±40 min

1. Cut out agarose gel bands corresponding to the 400–500 bp range (Figure 3D) and weigh no more than 300 mg. Transfer the gel slice into a sterile 1.5 mL Eppendorf tube using a clean, sharp scalpel.
   ▲ CRITICAL STEP: To prevent cross-contamination among multiple libraries, use a fresh scalpel for each librar.
2. Weigh the tube before and after cutting the gel to determine the weight of the gel slice accurately. Add three times the volume of Buffer QG relative to the weight of the gel slice (e.g., add 300 µL of Buffer QG for a 100 mg gel slice).
3. Place the tube on a rocking shaker at 50°C for at least 10 min until the gel fragment is completely dissolved. Incomplete dissolution will reduce the efficiency of DNA recovery. Briefly centrifuge the tube and let it stand at RT.
4. Add an equivalent volume of isopropanol to the gel solution (e.g., 100 µL of isopropanol for 100 mg of gel fragment), vortex thoroughly, and briefly centrifuge.
5. Transfer the gel-dissolving solution to a pre-warmed MiniElute Gel Extraction spin column. Centrifuge at 15,000 × *g* for 1 min at RT and discard the flow-through. If the total volume exceeds 700 μL, repeat this step with the remaining volume.
6. Add 500 μL of Buffer QG to the column. Let it sit for 2 min at RT and then centrifuge at 15,000 × *g* for 1 min.
7. Pipette 400 μL of Buffer PE into the column, incubate for 2 min, and then centrifuge at 15,000 × *g* for 1 min at RT and discard the flow-through. Repeat the washing step once more.
8. Centrifuge the columns again at 15,000 × *g* for 2 min at RT and carefully remove any residual buffer around the inner adsorption column using a 20 μL pipette tip.
9. Transfer the spin column into a sterile 1.5 mL Eppendorf tube and incubate the columns for 3–5 min at RT with the lid open to completely dry the membrane.
 10. Carefully pipette 15 μL of nuclease-free water preheated to 50°C (or Buffer EB) directly onto the center of the membrane and incubate at RT for 3 min with the lid closed.
11. Centrifuge for 2 min at 15,000 × *g* to collect the DNA fragments and measure the concentration with 1 μL of solution using a spectrophotometer.

**Table:** 

Component	Volume(μL)
KAPA HiFi HotStart Uracil + ReadyMix (2×)	12.5
PCR.primer.PE.1.0 (10 μM)	0.5
Multiplexing PCR Primer-Index N (10 μM)	0.5
Bisulfite-converted DNA	1
Nuclease-free water	10.5
Total	25

**Table:** 

Step	Temperature (°C)	Time	Numbers of cycle
Initial denaturation	95	2 min	1
Denaturation	95	15 s	12, 14 and 16
Annealing	65	40 s	
Extension	72	1 min	
Final extension	4	Hold	1

### 3.10 Recombination of the DNA fragments with the linearized PLB vector — ±20 min

▲ CRITICAL: The linearized PLB vector is convenient for recombinant construction and fragment sequencing due to its lethal gene which stops colonies from growing if this gene is not interrupted with the DNA insert. Taking into account the false positives, colony PCR with specific vector primers is a rapid screening strategy for the identification of positive recombinants and can be directly performed with colonies after lysing.

**FIGURE 5:**
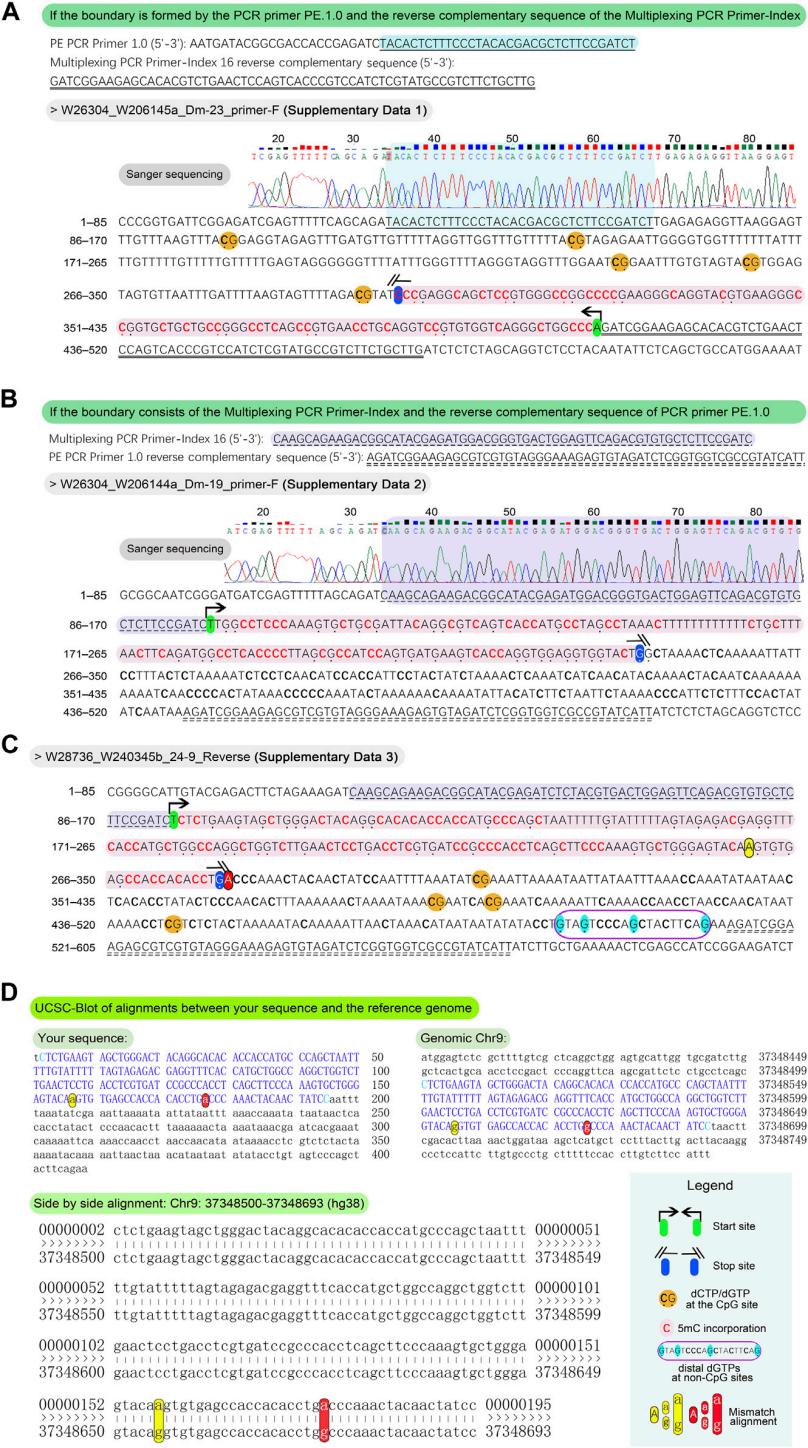
Methodology and examples for determination of the length of 5 mC incorporation. For the typical sequences, align the PCR primer PE.1.0 and the reverse complement of the Multiplexing PCR Primer-Index, or *vice versa*, to the outputs in Sanger sequencing fasta format. This alignment facilitates the delineation of DNA fragment boundaries for further analysis. The presence of bold “C” and emphasized “G,” along with highlighted sequences, aids in determining these boundaries. **(A)** If the boundary is formed by the PCR primer PE.1.0 and the reverse complementary sequence of the Multiplexing PCR Primer-Index, count the number of dNTPs from the 3′-end to the last dCTP at non-CpG sites (from the 3′ to 5′ direction) to determine the length of 5 mC incorporation. For example, a bold “C” at the 5′-end of a blue (not yellow) highlighted sequence marks the boundary, with an incorporation length of 109 bp. **(B)** If the boundary consists of the Multiplexing PCR Primer-Index and the reverse complement of the PCR primer PE.1.0, the number of dNTPs from the 5′-end to the last “G” at non-CpG sites (from the 5′ to 3′ direction) indicates the length of 5 mC incorporation. In the illustrated example, an emphasized “G” at the 5′-end of a blue highlighted sequence marks the boundary, with a length of 139 bp. To address sequences characterized by distal “C” or “G” at non-CpG sites, potentially influenced by sequencing artifacts or other unidentified factors, align the entire sequence to the reference genome utilizing the UCSC Blot tool. Exemplary alignment is illustrated in the figure, where four “Gs” at non-CpG sites are prominently displayed at the 3′-end, highlighted in cyan within a dark violet frame. **(C)** Based on the principle in the Figure 5B, the boundary of sequence in the example is determined as the last “G” at non-CpG sites, highlighted in blue, totaling 176 bp. For the removal of dNTPs at 3′-end exceeding 150 bp, the remaining length at the 5′-end of the DNA fragment must be > 150 bp to ensure sufficient acquisition of methylation data. In this instance, the remaining 233 bp at the 5′ end meet this criterion. **(D)** After the alignment of the full length of the output sequence to Chr9:37348500-37348693 (hg38) using the UCSC Blot tool (https://genome.ucsc.edu/cgi-bin/hgBlat?command=start), two mismatched non-CpG “a–g” pairs appear at the 3′ end, resulting from the bisulfite conversion of unmethylated “Cs.” This confirms the true boundary at the yellow highlighted “A,” with a length of 157 bp.

1. Perform the ligation reaction between the DNA fragments and the linearized PLB vector in a sterile 1.5 mL Eppendorf tube with the Lethal Based Fast Cloning Kit as shown below. Mix thoroughly and briefly centrifuge. Incubate the ligase reaction in an Eppendorf Thermomixer at 22°C for 5–15 min, and then place on ice.

2. Completely thaw DH5α competent cells on ice for 10 min and introduce them into the PLB ligation reaction at a ratio of 10:1 (vol/vol; e.g., 50 μL of competent cells for 5 μL of ligation solution). Gently mix by pipetting at least 10 times and maintain on ice for 30 min.
3. Subject the mixture to a heat shock at 42°C for 90 s, then immediately cool the tube on ice for 3 min. Under a laminar flow hood, add 500 μL of sterile LB medium and allow the cells to recover at 37°C with agitation at 150 rpm for 40 min.
  ▲ CRITICAL STEP: Handle much more gently to preserve the fragile electrostatic interactions between the DNA and the competent cell membrane.
4. Centrifuge at 800 × *g* for 3 min, discard 450 μL of the supernatant, resuspend the remaining 100 μL of cells, and then plate onto solid LB agar containing ampicillin (0.1 mg/mL). Incubate the plate at 37°C overnight in a constant temperature incubator.
5. Pick out a monoclonal colony with a 20 μL pipette tip and transfer it into 10 μL of sterile LB medium in a sterile 0.2 mL Eppendorf tube in a laminar flow cabinet. Dissolve the colony pellet completely by vortex vigorously and centrifuge briefly.
  ▲ CRITICAL STEP: Carefully extract the monoclonal colony from the solid LB medium to avoid disturbance that could lead to amplification of non-transformed cells and generate false positives.
6. Mix the colony PCR components in a sterile 0.2 mL Eppendorf tube as described below. If processing multiple samples, prepare a master mixture and dispense an aliquot to each reaction.

7. Perform PCR amplification according to the cycling conditions as follows:

 ▲ CRITICAL STEP: To prevent non-specific amplification, ensure the thermocycler is preheated to over 70°C before placing the tube, as Taq polymerase used here lacks a hot start capability.
8. Conduct electrophoresis of the libraries using a 2% agarose gel at 150 V for 30 min, and visualize under a UV transilluminator. The primers targeting the PLB vector (pLB vector forward sequencing primer and pLB vector reverse sequencing primer, refer to Supplementary Table S1) typically generate an amplification fragment of approximately 120 bp. Consequently, the PCR fragment size of the vector containing the insert is expected to range from 520 to 620 bp as illustrated in Figure 3E. It is essential to identify and sequence more than ten positive recombinants using the pLB vector forward or reverse sequencing primer.
9. Assess the length of 5 mC incorporation based on Sanger sequencing. Evaluate the sequencing results using the methodology illustrated in Figure 5, and examples employing examples provided in Supplementary Data S1–S3.
  ▲ CRITICAL The DNA library fragments amplified using 2 × KAPA HiFi HotStart Uracil + ReadyMix are blunt-ended, allowing the potential connection of both fragment ends to the same end of the linearized PLB vector. This configuration may result in distinct sequencing outputs when using either the pLB vector forward sequencing primer or the pLB vector reverse sequencing primer.
10. Amplify the final GPS libraries as described in Section 3.9, utilizing the optimal minimum number of PCR cycles which was pre-determined by the guiding length requirements from Sanger sequencing results. Perform a double size selection of the amplified library, targeting fragment sizes between 400 and 500 bp, using a 0.55/0.2 screening ratio of VAHTS DNA Clean Beads, as per the manufacturer’s instructions (BOX 3). Routinely elute in 22.5 μL of nuclease-free water to yield purified sequencing libraries of at least 40 ng. Detailed steps and practical tips for purification and enrichment are provided below. The preparation of magnetic beads and 80% alcohol is detailed in BOX 1. All processes are performed at RT.

**Table:** 

Reagent	Volume (μL)
2× Reaction Solution	2.5
PLB Vector (35 ng/μL)	0.5
DNA fragments	1.5
T4 DNA Ligase (3 U/µL)	0.5
Total	5

**Table:** 

Reagent	Volume (μL)
Colony-disolved LB	1
pLB Vector Forward Sequencing Primer (10 µM)	0.1
pLB Vector Reverse Sequencing Primer (10 µM)	0.1
2× Taq PCR Master Mix	5
Nuclease-free water	3.8
Total	10

**Table:** 

Step	Temperature (°C)	Time	Numbers of cycle
Initial denaturation	95	2 min	1
Denaturation	95	15 s	35
Annealing	58	40 s	
Extension	72	1 min	
Final extension	4	Hold	1

BOX 3 **Double size DNA selection strategy — ±45 min.**

Combine magnetic beads with DNA fragments exceeding the specified maximum length.

1. Measure the PCR products volume (25 μL) accurately and add 75 μL of nuclease-free water to achieve a total volume of 100 μL.
2. Thoroughly mix by pipetting up and down at least 20 times with a 200 μL pipette tip in a new sterile 1.5 mL Eppendorf tube, ensuring no bubbles form.
3. Add a 0.55× volume (55 μL for 100 μL of sample) of VAHTS DNA Clean Beads to the sample. Mix thoroughly by pipetting up and down at least 20 times.
4. Incubate the mixture at RT for 5 min to promote the binding of magnetic beads to DNA fragments longer than 500 bp.
5. Place the tube on a magnetic separation rack until the solution turns clear, typically for about 5 min.
6. Remove the supernatant carefully without disturbing the beads and transfer it to the bottom of a new sterile 1.5 mL Eppendorf tube held vertically, avoiding contact with the tube’s inner wall. Discard the beads.
  ▲ CRITICAL STEP: Avoid aspirating the magnetic beads, which could result in retention of overly long fragments in the final library. It is advisable to leave 1–2 μL of supernatant in the tube to prevent disturbing the beads, which minimally affects the final distribution range.
  ▲ CRITICAL STEP: Ensure the supernatant is not accidentally discarded. Marking the side of the tube can serve as a helpful reminder.
  Combination of magnetic beads and DNA fragments exceeding minimum distribution length.
7. Add 20 µL (0.2× volume) of VAHTS DNA Clean Beads to 155 µL of supernatant. Thoroughly mix the solution before placing the tube on the magnetic rack.
8. Incubate at RT for 5 min to enable binding of magnetic beads to DNA fragments longer than 400 bp.
9. Carefully aspirate and discard all supernatant, taking care not to disturb the beads.
10. Wash the beads twice with 200 µL of 80% ethanol, following the precise steps outlined in BOX 1. Allow the beads to air dry at RT.
11. Elute the dried beads with 23.5 µL of nuclease-free water. After 5 min of adsorption on the magnetic rack, transfer 22.5 µL of the DNA solution to a new sterile 1.5 mL Eppendorf tube.
12. Determine the library concentration using a Qubit 2.0 fluorometer with a Qubit dsDNA HS (High Sensitivity) Assay Kit, measuring 1 µL of the solution. The optimal concentration should be at least 2–3 ng/μL.
13. Assess the size distribution of the library by analyzing 1 µL of solution on an Agilent Bioanalyzer 2100 using High-Sensitivity DNA chips and reagents as per the manufacturer’s instructions. Refer to Figure 3F for library quality control details.
   ▲ CRITICAL STEP: Avoid aspirating the magnetic beads during the supernatant removal to maintain high recovery efficiency. Ensure all supernatant is discarded during steps in BOX 3-9 to prevent contamination with very short DNA fragments, which could interfere with the efficiency of GPS library amplification during sequencing.
   ▲ PAUSE POINT: The size-selected DNA should be stored at −20°C for up to 1 year or at −80°C for long-term storage.
  ◆ TROUBLESHOOTING

### 3.11 High-throughput DNA sequencing — ±7 days

Once the GPS library meets the qualification criteria, perform high-throughput sequencing to obtain 130 gigabases of 150 bp paired-end reads using the Illumina NovaSeq platforms. Set the cluster density to 20% relative to a non-bisulfite-sequencing library (e.g., RNA-seq or ChIP-seq). This cluster density is optimized to balance the CG-content, ensuring high-quality read data.

### 3.12 Data processing — ±1–3 days

▲CRITICAL: Following paired-end GPS sequencing, we obtained Read1 and Read2. According to the principles of GPS, after treatment with T4 DNA polymerase, unmethylated cytosines (Cs) in Read1 are converted to thymines (Ts) following bisulfite treatment and PCR amplification, while the cytosines in Read2 retain the original sequence of the reference genome. However, due to the complexity of the enzymatic reactions, the efficiency of T4 DNA polymerase varies among DNA fragments of different lengths, potentially leading to variable C→T conversion sequences in Read2, which may affect the alignment with the reference genome. To define the boundary, we have provided the fundamental analysis principles and illustrative examples in Figures 5A, B. For Read2, if T4 DNA polymerase adequately treated the 3′-end of the fragment, the Cs at the 3′-end would be fully methylated. Otherwise, the fragment may contain unmethylated Cs, which would be converted to Ts following bisulfite treatment (Figure 5A). In the complementary strand of Read2, C→T conversion corresponds to G→A conversion. Given that over 98% of DNA methylation in somatic cells occurs in a CpG dinucleotide context (Lister R et al., 2009), and the original methylation status is pending, we specifically used methylated non-CpG site in Read2 to determine the treatment boundary. Thus, we scanned Read2 from the 5′- to the 3′-end to locate the last 5′-[A/T/G]G-3′ sequence, which was designated as the boundary of T4 polymerase treatment (Figure 5B). The sequence from the 5′-end to this boundary was used for genomic alignment, with sequences shorter than 35 nt being discarded to eliminate non-specific alignment interference. Similarly, overtreatment by T4 polymerase may result in excessive methylation at the 3′-end of Read1. We scanned Read1 from the 3′- to the 5′-end to identify the first C-to-T conversion, which was defined as the boundary of T4 polymerase treatment. The sequence from the 5′-end to this boundary in Read1 was used for methylation analysis. For processing steps below, it is assumed that a Linux computing environment with all relevant software installed is available. The commands may be changed according to variable needs.
1.Sequencing quality control. Employ FastQC to assess the quality of high-throughput sequencing data. A typical command is:
*fastqc input_sequencin_data_R1.fastq.gz input_sequencin_data_R2.fastq.gz*
Further, use NGSQCToolkit to filter out poor quality reads and Illumina adapter sequences, thereby obtaining clean data. Use the command:
*IlluQC_PRLL.pl -pe sample_R1.fastq sample_R2.fastq -s 35 -L 70 -c 4*
   ▲ CRITICAL STEP: The “-l” parameter indicates that the percentage of qualified bases is over 70%, “-s” denotes the quality score threshold of 35, and “-c” sets the CPU count to 4.
2.Duplicate removal and conversion to fasta. Remove sequencing duplicates to enhance genome alignment efficiency. Convert the input fastq files to fasta format. In order to improve the genome alignment affection, we removing all the sequencing duplicates. The commands:
*$ sort -T input_data |uniq > outputfile*
*$ paste Read1.fastq Read2.fastq |awk “{if(NR%4 = = 2) print $0}”|sort -T ./ |uniq >*
*${input1}_step1*
*$filter_R2_by_CH_and_A_to_fasta.py ${input1}_step1*
   ▲ CRITICAL STEP: The “ filter_R2_by_CH_and_A_to_fasta.py ” is provided at Supplementary Data S4.
3.Single-end alignment of R2 reads. Perform alignment of the cleaned R2 reads to the reference genome using bowtie2 with the commands:
*bowtie2 -x reference_genome -f input_data -k 20 -p 25 -omit-sec-seq -S output.sam*
   ▲ CRITICAL STEP: The “-k” parameter allows reporting up to 20 alignments per read.
   ▲ TROUBLESHOOTING
4.R1 read positioning. Employ the Smith-Waterman algorithm to determine the position of R1 reads using the commands:
*$sw7.0 input_file reference_genome output_file score_matrix_n.txt score_matrix_p.txt 475 6 input3 input_unmap >> nohup.out.*
Then, identify R1 and R2 read pairs within a 1 kb region on the reference genome.
  ▲ CRITICAL STEP: The software “sw7.0” is available on GitHub at https://github.com/lijinbella/GPS. Scoring for the Smith-Waterman algorithm assigns 5 points for matching bases, 0 points for mismatches, and 6 points for indels.
   ▲ TROUBLESHOOTING
5.Read trimming: To maintain accurate methylation levels in GPS, trim R1 reads that are excessively cut and R2 reads that are insufficiently cut by T4 DNA polymerase. Use the command:
*mark_one_end_map_result.exe input1_out.sam input1_out.sam_mark_one_end -I -S*
   ▲ CRITICAL STEP: The software “mark_one_end_map_result.exe” can be obtained from GitHub at https://github.com/Luobajiang/GPS. The “-l” parameter sets the default read length to 100 bp; “-S” uses system time as the seed for the random number generator.
   ▲ TROUBLESHOOTING
6.Methylation calculation. Calculate the methylation level at each cytosine site, defined as the ratio of methylated cytosines (“C”) to the total of cytosines and thymines (“C” + “T”) at the same site in the reference genome.
7.The following steps outline the analysis procedure for genomic variation and combined DNA methylation analysis in allele-specific methylation patterns (ASMs) for reference.
  1.After completing steps 3.12.1 to 3.12.3, identify mutations and potential allele sites in the output.sam file using the script:
*allele_methy_1_report_mut-loc-cover.py output.sam*
     ▲ CRITICAL STEP: The script “allele_methy_1_report_mut-loc-cover.py” is available in Supplementary Data S5.
  2.For each potential allele site, categorize the SAM file into mutation-present and mutation-absent groups. Calculate the corresponding methylation levels in Read1 for each group.
*python ./allele_methy_get_have_and_no_goals_step3.py goal output.sam*
*python ./allele_methy_split_to_goal_step3_files.py output.sam_have_goal_step3*
*python ./allele_methy_split_to_goal_step3_files.py output.sam_no_goal_step3*
     ▲ CRITICAL STEP: The scripts “allele_methy_get_have_and_no_goals_step3.py” and “allele_methy_split_to_goal_step3_files.py” are provided in Supplementary Data S6, 7, respectively.
  3.Compare the methylation levels of each cytosine between mutation-present and mutation-absent groups. Based on the magnitude of methylation differences, generate a list of allele-specific methylation (methylation difference ≥ 70%, Read coverage ≥ 5):
*intersectBed -a goal_${i}_${type}.sam_have_goal_step3_sw1121out_human_methy_on_node_0.go -b goal_${i}_${type}.sam_no_goal_step3_sw1121out_human_methy_on_node_0.go -wo | awk “{if(sqrt(($5-$13)^2) >= 70) print $0}” | awk “{if(($7+$8) >= 5) print $0}” | awk “{if(($15+$16) >= 5) print $0}” >> report_have_dif70_cover5*

## 4 Anticipated results

We have successfully applied this protocol for constructing GPS libraries from normal liver cells, hepatoma cell line (Li et al., 2019), and five breast cell lines (BL. Zhang, W.Li, unpublished data). Based on our experience, the anticipated results include:

1. Fragment size optimization. The optimal fragment size for sonication ranges from 200 to 500 bp, with an average size of 300–350 bp (Figure 3B). Ligation of methylated adapters to both ends of the DNA fragments typically extends the size to 350–400 bp. Following library amplification and double size DNA selection, the size range of the final library is typically 400–500 bp (Figure 3D). It is advisable to verify any significant deviations in the size of DNA fragments.
2. DNA recovery yield. The expected yield ratios for DNA recovery post-reaction steps are detailed in Supplementary Table S2, which serves as a reference for quality control.
3. The length of 5 mC incorporation at 3′-end of DNA fragments. The removal length of dNTPs by T4 DNA polymerase should range from 36 to 150 bp (Figure 5). Lengths shorter than 36 bp are suboptimal for precise guidance of localization, whereas lengths exceeding 150 bp can diminish the yield of methylation information.
4. Final GPS Library PCR amplification. For the final GPS library amplification, the PCR typically reaches a plateau phase after 10 to 15 cycles, and after double size selection, the library fragments should range from 300 to 600 bp, with a predominant peak around 450 bp (Figure 5C), and the concentration usually ranges from 2 to 10 ng/μL in 20 µL of nuclease-free water, quantified using a Qubit fluorometer.
5. Sequencing coverage requirements. To achieve a minimum coverage of 5× across all cytosine sites in the genome, particularly in regions traditionally difficult to cover, we find that 150 bp paired-end sequencing with at least 130 gigabases of reads per library is adequate. In our published studies, the processed GPS library data typically show a minimum cytosine-to-uracil conversion efficiency of over 99%, coverage of unique CpG sites above 97%, and cytosine coverage exceeding 96%.
6. TROUBLESHOOTING advice can be found in Table 1.

**TABLE 1:** TROUBLESHOOTING advice.

Step	Problem	Possible reason	Solution
BOX 1-10	Cracks appear on the surface of the magnetic beads	The beads are dried out too much	Observe the reflectivity of the surface of the magnetic beads under the light
BOX 1-14	No or less recovery of DNA fraction	Inaccurate DNA quantification	Use Agilent High-Sensitivity DNA chips instead of spectophotometry
		The wrong volume ratio of DNA and magnetic beads	Ensure the correct volume ratio of DNA and magnetic beads according to the manufacturer’s instructions
		Poor or insufficient binding of VAHTS DNA Clean Beads	make sure the beads are at RT for at least 15 min prior to use
			Increase incubation time at RT.
		Poor-quality reagents (e.g., expired VAHTS DNA Clean Beads)	Check the VAHTS DNA Clean Beads expiry date. Aliquot fresh VAHTS DNA Clean Beads and store at 4°C to avoid repeat mixing
		The low concentration of wash buffer	Make sure the concentration of the wash buffer is 80% instead of 20% by testing its flammability
		Cracks appear on the surface of the magnetic beads	Do not allow the beads to dry out too much. When the surface of the magnetic beads is not reflective, add buffer to dissolve it
3.5.1-3	Insufficient removal of dNTPs	insufficient T4 DNA polymerase digestion time at 12°C	Increase incubation time at 12°C
		Inadequate Proteinase K digestion of DBPs	Check all reagents of Proteinase K digestion have been correctly dispensed and Increase digestion time
	Excessive removal of dNTPs	Excessive T4 DNA polymerase digestion time at 12°C	Quickly transfer the prepared sample from ice to 12°C and shorten incubation time
3.8.3-8	Low bisulfite conversion rate (< 99%)	Analysis parameter error	Check the bioinformatics analysis process and parameter settings
		Bisulfite conversion failed	Check the accuracy of the volume and concentration of all reagents and the incubation temperatures and time. Recommended to use the freshly prepared CT Conversion Reagent. Close the column lid tightly at step 3.8.3-6. Avoid pausing for a long time (<1 h) once the sodium bisulfite chemical reaction is started
3.9-4	No or low yield of the PCR amplified library	inadequate cycle number of pcr amplification	Increase 3–5 cycles of pcr amplification
		Poor PCR reagents quality	Ensure that the reagents for PCR have not expired. Make aliquots of the 2× KAPA HiFi HotStart Uracil + ReadyMix and the primers to avoid unnecessary freeze-thaw cycles
		Poor adapter ligation efficiency	Double the adapter ligation or dA-tailing reaction time individually or simultaneously
			Use fresh ligase reaction buffer containing undegraded ATP or add additional ATP to the reaction
		Low recovery of bisulfite conversion	Purify the DNA immediately after the thermal program is completed to avoid any potential DNA degradation
			Use buffer EB or nuclease-free water warmed to 50°C to facilitate elution from the column
	High yield of the PCR amplified library	excessive cycle number of PCR amplification	Reduce cycles of PCR amplification based on the brightness of the DNA Ladder
	Abnormal size/shape of the PCR amplified library	Insufficient/excessive sonication of DNA	Optimize sonication conditions for the DNA fragments ranging from 200 to 500 bp
		Excessive primer or adapter dimers contamination	Perform purification thoroughly before PCR amplification to completely eliminate the excess Adapter
			Repeat double size DNA selection for the post-purification PCR library
BOX 3-13	Extremely long fragments in the library	aspiration of the magnetic beads when transfer the supernatant at step BOX 3-6	Leave 1–2 μL of supernatant in the tube to avoid disturbing the beads at step BOX 3-6
	Extremely short fragments in the library	Supernatant residues caused by incomplete aspiration and washing at step BOX 3-9	Thoroughly aspirate and discard all of the supernatant at step BOX 3-9
3.12-3	R2 read could have many positions in the reference genome	R2 read could mapped to multiple locations on the reference genome by Bowtie2 algorithm	We give the parameters -k 20. The threshold is suitable for not only keeping enough R2 genome position information, but also locating R1 read sufficiently without causing more time and space
3.12-4	R1 read inaccurate location	When R2 was using to reach R1, the distance between R1 and R2 will affect the final pair of the two reads	After feature selection, we determined that when the distance between R1 and R2 read is 1Kilo bases, the R1-R2 position pair has the lowest false positive in the result
3.12-5	The base errors will be occurred by T4 DNA polymerase’s over-cutting or under-cutting on the read	The T4 polymerase has different restriction efficiency	The read trimming was added before the cytosine methylation calculation step. We judged the restriction site on the read, and cut or deleted the abnormal reads to ensure the accuracy of the next step

## 5 Conclusion, strengths, and limitations

In summary, the present protocol is an efficient way to rapidly and simultaneously obtain both DNA methylation and genetic variation data on a whole-genome scale. This is achieved by converting unmethylated cytosines to thymine at the R1 reads while preserving cytosines at the R2 reads unchanged post-bisulfite treatment. The advantages of GPS include:

1) The capability to simultaneously utilize the sequence of the 3′-end for genetic variation detection and the 5′-end sequence for genome-wide DNA methylation assessment, facilitating the preferential application of GPS for accurately detecting ASMs;
2) GPS demonstrates high efficiency, with approximately 80% of Read2 incorporating dNTPs containing 5 mC exceeding 100 bp by T4 DNA polymerase, and more than 96.5% treated to at least 50 bp, ensuring that Read2 precisely guides Read1 for accurate DNA methylation analysis;
3) Compared to traditional bisulfite sequencing (BS-seq), GPS facilitates more rapid and accurate alignment of bisulfite-converted sequences to the reference genome, attributable to the faster location-guiding effect of R2 reads which preserves the original base composition of the sequence;
4) GPS achieves cytosine coverage of up to 96%, with unbiased coverage in GC-rich and repetitive regions, improving the detection of repetitive sequences, CpG islands, and GC-rich areas, without distribution bias in promoters and other genomic functional elements (Li et al., 2019);
5) At equivalent sequencing costs, GPS provides much more reads with ≥5× CpG coverage, significantly enhancing productivity;
6) Method optimization has reduced the initial DNA input to as little as 100 ng, facilitating library construction from small cell and tissue samples;
7) GPS demonstrates stronger integration with other technologies, especially hybridization capture-BS-seq due to the preserve of original genome sequence.

In addition to the common limitation of BS-seq in failing to distinguish between 5-methylcytosine (5 mC) and 5-hydroxymethylcytosine (5hmC) (Houseman et al., 2016), the GPS has its own limitations, including:

1) Variations in GPS operation experience and enzyme batches can affect the precise removal of dNTPs during 5 mC incorporation;
2) GPS is not suited for extremely short or ultralow DNA samples, such as circulating free DNA. This protocol offers detailed guidance and practical tips to enhance experimental success, including optimizing T4 DNA polymerase reaction conditions and efficient DNA purification methods.

**FIGURE 6:**
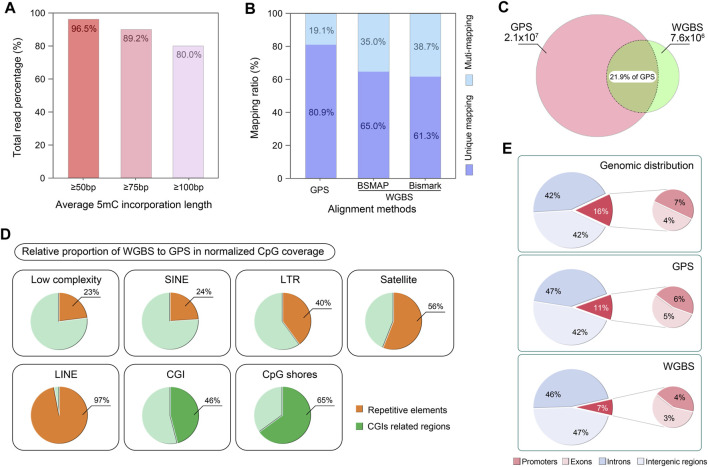
Comparison of methylation data derived from GPS and WGBS. **(A)** Leveraging the activity of T4 DNA polymerase, the GPS library is increasingly enriched with Read2 sequences containing 5 mC as their length increases. The graph shows that the percentages of Read2 sequences exceeding 50, 75, and 100 bp are 96.5%, 89.2%, and 80%, respectively. **(B)** Comparative analysis of alignment efficiency between GPS and WGBS for DNA methylation in 97L cells reveals that GPS achieves a unique mapped percentage of paired reads of 80.9%, which is 15%–20% higher than that obtained using the popular WGBS alignment tools, BSMAP and Bismark. **(C)** GPS identified more CpG sites at ≥ 5× coverage compared to WGBS at similar sequencing depths. Specifically, GPS detected 2.1 × 10^7^ CpG sites, while WGBS identified 7.6 × 10^6^ within a comparable dataset of bisulfite-converted data comprising 375M read. Both methods identified 4.6 × 10^6^ CpG sites, representing 21.9% of those detected by GPS, as indicated by the dashed circle in the figure. **(D)** GPS outperforms WGBS in detecting CpG sites within repetitive elements and CGIs related regions at similar sequencing depths. The *y*-axis represents the percentage ratio of CpG sites covered by WGBS relative to GPS. The *x*-axis showing Low complexity, short interspersed nuclear element (SINE), Long Terminal Repeat (LTR), Satellite, long interspersed nuclear element (LINE), CGI, and CpG shores, respectively. **(E)** Comparative analyses of CpG site distributions across promoter, exon, intron, and intergenic regions reveal that GPS more accurately reflects the genomic distribution of CpG sites than WGBS, with notable fidelity in promoter and exon regions.

Compared to other protocols, we believe that our protocol offers a unique combination of advantages. We have conducted a comprehensive comparison between GPS and WGBS in 97L hepatocellular carcinoma cells, considering various parameters such as high efficiency of Read2 incorporating dNTPs containing 5 mC (Figure 6A), alignment efficiency (Figure 6B), methylation profiling output at ≥5× coverage (Figure 6C), CpG sites within repetitive elements and CGI-related regions (Figure 6D), distributions of CpG sites across promoters, exons, introns, and intergenic regions (Figure 6E). GPS demonstrated marked advantages over WGBS in the aspect mentioned above (Please refer to Figure 6 for specific comparison results). In DNA methylation profiling of normal human liver cells, GPS exhibited a coverage rate of 97% for CpG sites and 96% for cytosine sites, significantly outperforming WGBS, which typically achieved coverage of approximately 90% of all CpG sites. Additionally, cost estimates suggest that the overall expense of employing GPS is approximately 70% of that for WGBS, with the average cost per million covered CpG sites ranging between $10 and $12. In terms of detecting genetic variants, GPS surpassed WGBS, identifying more variants with the same raw reads. Notably, 91% of the 2,296,462 variations found in liver cells by GPS matched entries in the dbSNP database. This efficacy demonstrates GPS’s ability to capture both genetic and epigenetic data in a single assay, enhancing the exploration of interactions such as ASM. Specifically, GPS detected 1,820 ASMs, substantially more than the 135 ASMs identified by WGBS with an equivalent dataset (Li et al., 2019).

## Data Availability

The GPS data analyzed in this study are from a previous study (Li et al., 2019) in NCBI’s Gene Expression Omnibus (GEO; http://www.ncbi.nlm.nih.gov/geo/) and are accessible through GEO Series accession number GSE92328. The software tools applied in this article, ‘sw7.0’ and ‘mark_one_end_map_result.exe’, are available on GitHub at https://github.com/lijinbella/GPS and https://github.com/Luobajiang/GPS, respectively. The scripts utilized in the bioinformatics analysis, ‘filter_R2_by_CH_and_A_to_fasta.py’, ‘allele_methy_1_report_mut-loc-cover.py’, ‘allele_methy_get_have_and_no_goals_step3.py’, and ‘allele_methy_split_to_goal_step3_files.py’, are provided in Supplementary Data S4–S7 of Supplementary Material, respectively. Further inquiries can be directed to the corresponding author.
