# Supplementary material for: Rapid genome-wide profiling of DNA methylation and genetic variation using guide positioning sequencing (GPS)
Source: Front Cell Dev Biol. 2024 Sep 24;12:1457387. doi: 10.3389/fcell.2024.1457387 (PMC11459621; doi:10.3389/fcell.2024.1457387)
Supplement: Supplementary file 1 [file DataSheet1.doc]

**Supplementary Data and Tables for Rapid Genome-wide Profiling of DNA Methylation and Genetic Variation Using Guide Positioning Sequencing (GPS)**

Baolong Zhang, Wei Li, Jin Li, Yan Li, Huaibing Luo, Yanping Xi, Shihua Dong, Feizhen Wu and Wenqiang Yu.

**Supplementary Data**

**Supplementary Data 1 W26304_W206145a_Dm-23_primer-F.seq**

CCCGGTGATTCGGAGATCGAGTTTTTCAGCAGATACACTCTTTCCCTACACGACGCTCTTCCGATCTTGAGAGAGGTTAAGGAGTTTGTTTAAGTTTACGGAGGTAGAGTTTGATGTTGTTTTTAGGTTGGTTTGTTTTTACGTAGAGAATTGGGGTGGTTTTTTTATTTTTGTTTTTGTTTTTGTTTTTGAGTAGGGGGGTTTTATTTGGGTTTTAGGGTAGGTTTGGAATCGGAATTTGTGTAGTACGTGGAGTAGTGTTAATTTGATTTTAAGTAGTTTTAGACGTATCCCGAGGCAGCTCCGTGGGCCGGCCCCGAAGGGCAGGTACGTGAAGGGCCGGTGCTGCTGCCGGGCCTCAGCCGTGAACCTGCAGGTCCGTGTGGTCAGGGCTGGCCCAGATCGGAAGAGCACACGTCTGAACTCCAGTCACCCGTCCATCTCGTATGCCGTCTTCTGCTTGATCTCTCTAGCAGGTCTCCTACAATATTCTCAGCTGCCATGGAAAATCGATGTTCTTCTTTTATTCTCTCAAGATTTTCAGGCTGTATATTAAAACTTATATTAAGAACTATGCTAACCACCTCATCAGGAACCGTTGTAGGTGGCGTGGGTTTTCTTGGCAATCGACTCTCATGAAAACTACGAGCTAAATATTCAATATGTTCCTCTTGACCAACTTTATTCTGCATTTTTTTTGAACGAGGTTTAGAGCAAGCTTCAGGAAACTGAGACAGGAATTTTATTAAAAATTTAAATTTGAAGAAAGTTCAGGGTTAATAGCATCCATTTTTTGCTTTGCAAGTTCCTCAGCATTCTTAACAAAGACGTCTCTTTTGACATGTTTAAAGTTTAAACCTCCTGTGTGAAATTATTATCCGCTCATATTTCCACACCTTATACTAGCCGAAGCATAAAGTGTAAGCCTGGGTGCCTAATGAGTGAGCTAACTCACATTAGATTGCGTTGGGTCCCTGCCAATTG

**Supplementary Data 2 W26304_W206144a_Dm-19_primer-F.seq**

GCGGCAATCGGGATGATCGAGTTTTTAGCAGATCAAGCAGAAGACGGCATACGAGATGGACGGGTGACTGGAGTTCAGACGTGTGCTCTTCCGATCTTGGCCTCCCAAAGTGCTGCGATTACAGGCGTCAGTCACCATGCCTAGCCTAAACTTTTTTTTTTTTCTGCTTTAACTTCAGATGGCCTCACCCCTTAGCGCCATCCAGTGATGAAGTCACCAGGTGGAGGTGGTACTGGCTAAAACTCAAAAATTATTCCTTTACTCTAAAAATCTCCTCAACATCCACCATTCCTACTATCTAAAACTCAAATCATCAACATACAAAACTACAATCAAAAAAAAAATCAACCCCACTATAAACCCCCAAATACTAAAAAACAAAATATTACATCTTCTAATTCTAAAACCCATTCTCTTTCCACTATATCAATAAAGATCGGAAGAGCGTCGTGTAGGGAAAGAGTGTAGATCTCGGTGGTCGCCGTATCATTATCTCTCTAGCAGGTCTCCTACAATATTCTCAGCTGCCATGGAAAATCGATGTTCTTCTTTTATTCTCTCAAGATTTTCAGGCTGTATATTAAAACTTATATTAAGAACTATGCTAACCACCTCATCAGGAACCGTTGTAGGTGGCGTGGGTTTTCTTGGCAATCGACTCTCATGAAAACTACGAGCTAAATATTCAATATGTTCCTCTTGACCAACTTTATTCTGCATTTTTTTTGAACGAGGTTTAGAGCAAGCTTCAGGAAACTGAGACAGGAATTTTATTAAAAATTTAAATTTTGAAGAAAGTTCAGGGTTAATAGCATCCATTTTTTGCTTTGCAAGTTCCTCAGCATTCTTAACAAAAGACGTCTCTTTTGACATGTTTAAAGTTTAAACCTCCTGTGTGAAATTATTATCCGCTCATAATTCCACACATTATACGAGCCGGAAGCATAAAGTGTAAAGCCTGGGGTGCCTAATGAGTGAGCTAACTCACATTAATTGCGTT

**Supplementary Data 3 W28736_W240345b_24-9_Reverse.seq**

CGGGGCATTGTACGAGACTTCTAGAAAGATCAAGCAGAAGACGGCATACGAGATCTCTACGTGACTGGAGTTCAGACGTGTGCTCTTCCGATCTCTCTGAAGTAGCTGGGACTACAGGCACACACCACCATGCCCAGCTAATTTTTGTATTTTTAGTAGAGACGAGGTTTCACCATGCTGGCCAGGCTGGTCTTGAACTCCTGACCTCGTGATCCGCCCACCTCAGCTTCCCAAAGTGCTGGGAGTACAAGTGTGAGCCACCACACCTGACCCAAACTACAACTATCCAATTTTAAATATCGAAATTAAAATAATTATAATTTAAACCAAATATAATAACTCACACCTATACTCCCAACACTTTAAAAAACTAAAATAAACGAATCACGAAATCAAAAATTCAAAACCAACCTAACCAACATAATAAAACCTCGTCTCTACTAAAAATACAAAAATTAACTAAACATAATAATATATACCTGTAGTCCCAGCTACTTCAGAAAGATCGGAAGAGCGTCGTGTAGGGAAAGAGTGTAGATCTCGGTGGTCGCCGTATCATTATCTTGCTGAAAAACTCGAGCCATCCGGAAGATCTGGCGGCCGCTCTCCCTATAGTGAGTCGTATTACGCCGGATGGATATGGTGTTCAGGCACAAGTGTTAAAGCAGTTGATTTTATTCACTATGATGAAAAAAACAATGAATGGAACCTGCTCCAAGTTAAAAATAGAGATAATACCGAAAACTCATCGAGTAGTAAGATTAGAGATAATACAACAATAAAAAAATGGTTTAGAACTTACTCACAGCGTGATGCTACTAATTGGGACAATTTTCCAGATGAAGTATCATCTAAGAATTTAAATGAAGAAGACTTCAGAGCTTTTGTTAAAAATTATTTGGCAAAAATAATATAATTCGGCTGCAGGGGCGGCCTCGTGATACGCCTATTTTTATAGGTTAATGTCATGATAATAATGGTTTTCTTAGACGTCAAGTGG

**Supplementary Data 4 filter_R2_by_CH_and_A_to_fasta.py**

#!/usr/bin/python

# -*- coding: utf-8 -*-

import sys

import re

def main():

if len(sys.argv) == 2:

step1_uniq_file = sys.argv[1]

else:

print "please input step1_uniq file"

sys.exit(1)

out_file = str(step1_uniq_file)+"2"

fin = open(step1_uniq_file, 'rU')

fout = open(out_file,'w')

i = 0

for line in fin:

i+=1

line = line.strip()

eles = line.split()

r1_seq = eles[0]

r2_seq = eles[1]

#substrs = re.search('TACAAG',r1_seq, flags = re.IGNORECASE)

# boundary = r1_seq.find('TACAAG')+12

# print boundary

# r1_seq_cut = r1_seq[boundary:29]

# print r1_seq_cut

# print >>fout, '%s%d%s' %(">",i,"_")

# print >>fout, '%s' %(r1_seq_cut)

# substrs = re.findall('[atg]g', r2_seq, flags = re.IGNORECASE)

# if not substrs :

# pass

# else:

# boundary = r2_seq.rfind(substrs[-1]) + 1

# if boundary >35:

# r2_seq_cut = r2_seq[0:boundary]

# print >>fout, '%s%d%s%s' %(">",i,"_",r1_seq)

# print >>fout, '%s' %(r2_seq_cut)

substrs = re.findall('[agt]g', r2_seq, flags = re.IGNORECASE)

if not substrs:

substrs_A = r2_seq.find('A')

if substrs_A == -1:

boundary = 100

else:

boundary = substrs_A

else:

boundary_tem = r2_seq.rfind(substrs[-1]) + 2

left_length = r2_seq[boundary_tem:]

re_findA = left_length.find('A')

boundary = boundary_tem + re_findA

if boundary >= 35:

r2_seq_cut = r2_seq[0:boundary]

print >>fout, '%s%d%s%s' %(">",i,"_",r1_seq)

print >>fout, '%s' %(r2_seq_cut)

fout.close()

if __name__ == '__main__':

main()

**Supplementary Data 5 allele_methy_1_report_mut-loc-cover.py**

#!/usr/bin/python

# -*- coding: utf-8 -*-

import sys

import re

def main():

chr_hash = {'chr1':{}, 'chr2':{}, 'chr3':{}, 'chr4':{}, 'chr5':{}, 'chr6':{},

'chr7':{}, 'chr8':{}, 'chr9':{}, 'chr10':{}, 'chr11':{}, 'chr12':{}, 'chr13':{},

'chr14':{}, 'chr15':{}, 'chr16':{}, 'chr17':{}, 'chr18':{}, 'chr19':{}, 'chr20':{}, 'chr21':{}, 'chr22':{}, 'chrX':{}, 'chrY':{}, 'chrM':{}}

#sam_file = '/home/lijin/data/allele_specific_methylation/python/test_sam'

#out_file = '/home/lijin/data/allele_specific_methylation/python/test_samwoooo'

if len(sys.argv) == 2:

sam_file = sys.argv[1]

else:

print "please input sam file"

sys.exit(1)

out_file = str(sam_file) + ".mut-loc-cover"

fin = open(sam_file, 'rU')

line_readed = 1

for line in fin:

if line_readed % 1000000 == 0:

print '%d reads have been readed' %line_readed

line_readed += 1

line = line.strip()

eles = line.split()

readid = eles[0]

strand = eles[1]

chrm = eles[2]

if chrm not in chr_hash:

print "chromsome: %s not in chr_hash" %chrm

sys.exit(1)

start = int(eles[3])

seq = eles[4]

if len(eles) == 6:

mut_str = eles[5]

else:

mut_str = ""

if strand =="+":

substrs = re.findall('[atg]g',seq, flags = re.IGNORECASE)

if not substrs:

boundary = -1

else:

boundary = seq.rfind(substrs[-1]) + 1

mut_list = mut_str.split(',')

for mut in mut_list:

if not mut:

pass

else:

mut_eles = mut.split(":")

mut_pos = int(mut_eles[0])

mut_pattern = mut_eles[1]

if mut_pos < boundary or mut_pattern != 'G>A':

mut_loci = start + mut_pos

if mut_loci in chr_hash[chrm]:

chr_hash[chrm][mut_loci] += 1

else:

chr_hash[chrm][mut_loci] = 1

else:

substrs = re.search('c[atc]',seq, flags = re.IGNORECASE)

if not substrs:

boundary = -1

else:

boundary = 99-seq.find(substrs.group(0))

mut_list = mut_str.split(',')

for mut in mut_list:

if not mut:

pass

else:

mut_eles = mut.split(":")

mut_pos = int(mut_eles[0])

mut_pattern = mut_eles[1]

if mut_pos < boundary or mut_pattern != 'C>T':

mut_loci = start - mut_pos + 99

if mut_loci in chr_hash[chrm]:

chr_hash[chrm][mut_loci] += 1

else:

chr_hash[chrm][mut_loci] = 1

fin.close()

fout = open(out_file,'w')

for key,val in chr_hash.iteritems():

chrm = key

for mut_loci, read_count in val.iteritems():

mut_loci_plus1 = mut_loci + 1

print >> fout, '%s\t%d\t%d\t%d' %(chrm, mut_loci,mut_loci_plus1, read_count)

fout.close()

if __name__ == '__main__':

main()

**Supplementary Data 6 allele_methy_get_have_and_no_goals_step3.py**

#!/usr/bin/python

# -*- coding: utf-8 -*-

import sys

import re

def main():

if len(sys.argv) == 3:

goal_file = sys.argv[1]

sam_file = sys.argv[2]

else:

print "please input goal and sam file"

sys.exit(1)

out_file_no_goal = str(sam_file) + "_no_" + str(goal_file) + "_step3"

fout_no_goal = open(out_file_no_goal, 'w')

out_file_have_goal = str(sam_file) + "_have_" + str(goal_file) + "_step3"

fout_have_goal = open(out_file_have_goal, 'w')

#fin_goal = open(goal_file, 'rU')

fin_sam = open(sam_file, 'rU')

goal_list = []

fin_goal = open(goal_file, 'rU')

for goal_line in fin_goal:

goal_line = goal_line.strip()

goal_eles = goal_line.split()

goal_list.append((goal_eles[0],int(goal_eles[1])))

fin_goal.close()

line_readed = 1

for line in fin_sam:

if line_readed % 1000000 == 0:

print '%d sam lines readed' %line_readed

line_readed += 1

sam_line = line.strip()

eles = sam_line.split()

readid = eles[0]

strand = eles[1]

chrm = eles[2]

start = int(eles[3])

seq = eles[4]

if len(eles) == 6:

mut_str = eles[5]

else:

mut_str = ""

fin_goal = open(goal_file, 'rU')

goal_line_readed = 1

goal_num = 1

for goal_ele in goal_list:

goal_chrm = goal_ele[0]

goal_start = goal_ele[1]

goal_mark = "goal_" + str(goal_num)

goal_num += 1

distance = goal_start - start

if distance >=0 and distance <=99 and goal_chrm == chrm:

if mut_str == "":

print >> fout_no_goal, '%s\t%d\t%s\t%s\t%s\t%s\t%d' %(goal_chrm, goal_start,readid,goal_mark, strand, chrm, start)

else:

a = 0

mut_list = mut_str.split(',')

for mut in mut_list:

mut_eles = mut.split(":")

mut_pos = int(mut_eles[0])

mut_pattern = mut_eles[1]

if strand == "+":

mut_start = start + mut_pos

else:

mut_start = start + 99 - mut_pos

if goal_start == mut_start:

a += 1

if strand =="+":

substrs = re.findall('[atg]g',seq, flags = re.IGNORECASE)

if not substrs:

boundary = -1

else:

boundary = seq.rfind(substrs[-1]) + 1

if mut_pos < boundary or mut_pattern != 'G>A':

print >> fout_have_goal, '%s\t%d\t%s\t%s\t%s\t%s\t%s' %(goal_chrm, goal_start,readid, goal_mark, strand, chrm, start)

else:

pass

else:

substrs = re.search('c[atc]',seq, flags = re.IGNORECASE)

if not substrs:

boundary = -1

else:

boundary = 99-seq.find(substrs.group(0))

if mut_pos < boundary or mut_pattern != 'C>T':

print >> fout_have_goal, '%s\t%d\t%s\t%s\t%s\t%s\t%s' %(goal_chrm, goal_start,readid, goal_mark, strand, chrm, start)

else:

pass

#if XXboundary:

#print >> fout_have_goal, '%s\t%s\t%s\t%s\t%s' %(readid, mut_pattern, strand, chrm, start)

#else:

#pass

else:

pass

if a == 0:

print >> fout_no_goal, '%s\t%d\t%s\t%s\t%s\t%s\t%d' %(goal_chrm, goal_start,readid,goal_mark, strand, chrm, start)

else:

pass

else:

pass

fin_goal.close()

fin_sam.close()

fout_have_goal.close()

fout_no_goal.close()

if __name__ == '__main__':

main()

**Supplementary Data 7 allele_methy_split_to_goal_step3_files.py**

#!/usr/bin/python

# -*- coding: utf-8 -*-

import sys

import re

import os

def main():

if len(sys.argv) == 2:

step3_file = sys.argv[1]

else:

print "please input step3 file"

sys.exit(1)

os.system('rm goal*')

fin = open(step3_file, 'rU')

line_readed = 1

for line in fin:

if line_readed % 1000000 == 0:

print '%d reads have been readed' %line_readed

line_readed += 1

line = line.strip()

eles = line.split()

goal_id = eles[3]

readid = eles[2]

strand = eles[4]

chrm = eles[5]

start = int(eles[6])

goal_file = str(goal_id)+"_"+str(step3_file)

fout = open(goal_file, 'a')

print >>fout, "%s\t%s\t%s\t%s\t%d" %(readid,"*",strand, chrm, start)

fin.close()

fout.close()

if __name__ == '__main__':

main()

**Supplementary Tables**

**Supplementary Table 1 | Details of the oligonucleotides used in this protocol**

| **Oligo name** | **Sequence (5'-3')** | **Length (nt)** | **Index sequence** |
| --- | --- | --- | --- |
| Multiplexing Adapters-as-methyC-R1 | CCTACACGACGCTCTTCCGATCt[C=5mC] | 23 | NA |
| Multiplexing Adapters-NH2-methyC-R2 | [Phos]GATCGGAAGAGCACACGTCT[Amino] [C=5mC] | 20 | NA |
| PCR. primer. PE.1.0 | AATGATACGGCGACCACCGAGATCTACACTCTTTCCCTACACGACGCTCTTCCGATCt | 58 | NA |
| Multiplexing PCR Primer-Index 1 | CAAGCAGAAGACGGCATACGAGATCGTGATGTGACTGGAGTTCAGACGTGTGCTCTTCCGATCT | 64 | ATCACG |
| Multiplexing PCR Primer-Index 2 | CAAGCAGAAGACGGCATACGAGATACATCGGTGACTGGAGTTCAGACGTGTGCTCTTCCGATCT | 64 | CGATGT |
| Multiplexing PCR Primer-Index3 | CAAGCAGAAGACGGCATACGAGATGCCTAAGTGACTGGAGTTCAGACGTGTGCTCTTCCGATCT | 64 | TTAGGC |
| Multiplexing PCR Primer-Index4 | CAAGCAGAAGACGGCATACGAGATTGGTCAGTGACTGGAGTTCAGACGTGTGCTCTTCCGATCT | 64 | TGACCA |
| Multiplexing PCR Primer-Index5 | CAAGCAGAAGACGGCATACGAGATCACTGTGTGACTGGAGTTCAGACGTGTGCTCTTCCGATCT | 64 | ACAGTG |
| Multiplexing PCR Primer-Index6 | CAAGCAGAAGACGGCATACGAGATATTGGCGTGACTGGAGTTCAGACGTGTGCTCTTCCGATCT | 64 | GCCAAT |
| Multiplexing PCR Primer-Index7 | CAAGCAGAAGACGGCATACGAGATGATCTGGTGACTGGAGTTCAGACGTGTGCTCTTCCGATCT | 64 | CAGATC |
| Multiplexing PCR Primer-Index8 | CAAGCAGAAGACGGCATACGAGATTCAAGTGTGACTGGAGTTCAGACGTGTGCTCTTCCGATCT | 64 | ACTTGA |
| Multiplexing PCR Primer-Index9 | CAAGCAGAAGACGGCATACGAGATCTGATCGTGACTGGAGTTCAGACGTGTGCTCTTCCGATCT | 64 | GATCAG |
| Multiplexing PCR Primer-Index10 | CAAGCAGAAGACGGCATACGAGATAAGCTAGTGACTGGAGTTCAGACGTGTGCTCTTCCGATCT | 64 | TAGCTT |
| Multiplexing PCR Primer-Index11 | CAAGCAGAAGACGGCATACGAGATGTAGCCGTGACTGGAGTTCAGACGTGTGCTCTTCCGATCT | 64 | GGCTAC |
| Multiplexing PCR Primer-Index12 | CAAGCAGAAGACGGCATACGAGATTACAAGGTGACTGGAGTTCAGACGTGTGCTCTTCCGATCT | 64 | CTTGTA |
| Multiplexing PCR Primer-Index13 | CAAGCAGAAGACGGCATACGAGATTTGACTGTGACTGGAGTTCAGACGTGTGCTCTTCCGATCT | 64 | AGTCAA |
| Multiplexing PCR Primer-Index14 | CAAGCAGAAGACGGCATACGAGATGGAACTGTGACTGGAGTTCAGACGTGTGCTCTTCCGATCT | 64 | AGTTCC |
| Multiplexing PCR Primer-Index15 | CAAGCAGAAGACGGCATACGAGATTGACATGTGACTGGAGTTCAGACGTGTGCTCTTCCGATCT | 64 | ATGTCA |
| Multiplexing PCR Primer-Index16 | CAAGCAGAAGACGGCATACGAGATGGACGGGTGACTGGAGTTCAGACGTGTGCTCTTCCGATCT | 64 | CCGTCC |
| Multiplexing PCR Primer-Index17 | CAAGCAGAAGACGGCATACGAGATCTCTACGTGACTGGAGTTCAGACGTGTGCTCTTCCGATCT | 64 | GTAGAG |
| Multiplexing PCR Primer-Index18 | CAAGCAGAAGACGGCATACGAGATGCGGACGTGACTGGAGTTCAGACGTGTGCTCTTCCGATCT | 64 | GTCCGC |
| pLB Sequencing Forward Primer | CGACTCACTATAGGGAGAGCGGC | 23 | NA |
| pLB Sequencing Reverse Primer | AAGAACATCGATTTTCCATGGCAG | 24 | NA |

[C=5mC]: All dCTPs in adapter oligonucleotides undergo methylated modification.

[Phos] and [Amino]: Additional modifications for Multiplexing Adapters-NH2-methyC-R2 include adding a phosphate group at the 5'-end and an amino group at the 3'-end.

**Supplementary Table 2 | Summary of the recovery ratio of DNA purification**

| **Steps** | **Reactions** | **Recovery ratio** |
| --- | --- | --- |
| **Step 3.3-3** | DNA sonication and End repair | ~ 50-60% |
| **Step 3.4-2** | Proteinase K digestion | **~** 95-100% |
| **Step 3.6-4** | dA-tailing reaction | ~ 50-60% |
| **Step 3.7-5** | Adapter ligation | **~** 95-100% |
